# Accuracy of Large Language Models for Literature Screening in Thoracic Surgery: Diagnostic Study

**DOI:** 10.2196/67488

**Published:** 2025-03-11

**Authors:** Zhang-Yi Dai, Fu-Qiang Wang, Cheng Shen, Yan-Li Ji, Zhi-Yang Li, Yun Wang, Qiang Pu

**Affiliations:** 1 Department of Thoracic Surgery West China Hospital of Sichuan University Chengdu China

**Keywords:** accuracy, large language models, meta-analysis, literature screening, thoracic surgery

## Abstract

**Background:**

Systematic reviews and meta-analyses rely on labor-intensive literature screening. While machine learning offers potential automation, its accuracy remains suboptimal. This raises the question of whether emerging large language models (LLMs) can provide a more accurate and efficient approach.

**Objective:**

This paper evaluates the sensitivity, specificity, and summary receiver operating characteristic (SROC) curve of LLM-assisted literature screening.

**Methods:**

We conducted a diagnostic study comparing the accuracy of LLM-assisted screening versus manual literature screening across 6 thoracic surgery meta-analyses. Manual screening by 2 investigators served as the reference standard. LLM-assisted screening was performed using ChatGPT-4o (OpenAI) and Claude-3.5 (Anthropic) sonnet, with discrepancies resolved by Gemini-1.5 pro (Google). In addition, 2 open-source, machine learning–based screening tools, ASReview (Utrecht University) and Abstrackr (Center for Evidence Synthesis in Health, Brown University School of Public Health), were also evaluated. We calculated sensitivity, specificity, and 95% CIs for the title and abstract, as well as full-text screening, generating pooled estimates and SROC curves. LLM prompts were revised based on a post hoc error analysis.

**Results:**

LLM-assisted full-text screening demonstrated high pooled sensitivity (0.87, 95% CI 0.77-0.99) and specificity (0.96, 95% CI 0.91-0.98), with the area under the curve (AUC) of 0.96 (95% CI 0.94-0.97). Title and abstract screening achieved a pooled sensitivity of 0.73 (95% CI 0.57-0.85) and specificity of 0.99 (95% CI 0.97-0.99), with an AUC of 0.97 (95% CI 0.96-0.99). Post hoc revisions improved sensitivity to 0.98 (95% CI 0.74-1.00) while maintaining high specificity (0.98, 95% CI 0.94-0.99). In comparison, the pooled sensitivity and specificity of ASReview tool-assisted screening were 0.58 (95% CI 0.53-0.64) and 0.97 (95% CI 0.91-0.99), respectively, with an AUC of 0.66 (95% CI 0.62-0.70). The pooled sensitivity and specificity of Abstrackr tool-assisted screening were 0.48 (95% CI 0.35-0.62) and 0.96 (95% CI 0.88-0.99), respectively, with an AUC of 0.78 (95% CI 0.74-0.82). A post hoc meta-analysis revealed comparable effect sizes between LLM-assisted and conventional screening.

**Conclusions:**

LLMs hold significant potential for streamlining literature screening in systematic reviews, reducing workload without sacrificing quality. Importantly, LLMs outperformed traditional machine learning-based tools (ASReview and Abstrackr) in both sensitivity and AUC values, suggesting that LLMs offer a more accurate and efficient approach to literature screening.

## Introduction

The development of clinical practice guidelines necessitates a comprehensive and systematic synthesis of current research evidence [1]. Evidence-based medicine frequently relies on systematic reviews and meta-analyses, which aggregate findings from studies on a specific topic [2-4]. This process traditionally involves extensive effort in identifying and retrieving relevant literature [5,6]. While machine learning has shown promise in streamlining literature retrieval, its accuracy often falls short of desired standards [7-9]. Therefore, further research is needed to develop a more precise screening method.

Recently, large language models (LLMs) powered by natural language processing have demonstrated remarkable capabilities in various domains, including language comprehension, image and video generation, and data analysis [8-13]. Previous studies have suggested the potential of LLMs for literature screening [9,14,15]. However, their accuracy in the screening process for meta-analyses remains unclear.

We hypothesized that LLM-assisted literature screening could achieve accuracy comparable with manual screening. To test this hypothesis, we designed a diagnostic trial using conventional manual screening as the reference standard to evaluate the accuracy of LLM-assisted literature screening.

## Methods

### Study Design

This prospective diagnostic study aimed to assess the validity of LLMs for assisting with literature screening during meta-analysis. Conventional literature manual screening served as the reference standard. This diagnostic study was performed according to the Standards for Reporting of Diagnostic Accuracy Studies (STARD) guidelines and the CONSORT-EHEALTH (Consolidated Standards of Reporting Trials of Electronic and Mobile Health Applications and Online Telehealth) checklist.

Before the study commenced, we defined the research topic as the comparison between sublobar resection and lobectomy in thoracic surgery, a topic of ongoing debate within the field. A total of 6 relevant published meta-analyses were identified [16-21], and the search strategy and terms were subsequently redesigned for a new round of literature retrieval and screening. To ensure a comprehensive yet nonredundant literature base, the search strategies and inclusion timeframes outlined within each meta-analysis were replicated. Duplicate studies were subsequently identified and removed using Rayyan [22], a web-based literature management tool.

### Conventional Literature Screening

Following identification, 2 independent investigators (Lei Peng and Xing-Yu Liu) screened the titles and abstracts of retrieved studies for inclusion based on predefined criteria (detailed in Table S1 in Multimedia Appendix 1). Discrepancies were resolved through adjudication by a third investigator (Xu-Yang Wang). Full-text papers of potentially eligible studies were subsequently reviewed by the same 2 independent investigators (Lei Peng and Xing-Yu Liu) against the same inclusion criteria, and any discrepancies were again resolved through adjudication by the third investigator (Xu-Yang Wang). This conventional literature screening established the reference standard for comparison. Investigators involved in the conventional screening were excluded from participation in the LLM-assisted screening and subsequent analyses.

### LLM-Assisted Literature Screening

For the LLM-assisted literature screening, a 5-column table (author, publication year, journal, title, and abstract) was compiled from the deduplicated literature. Following established prompt engineering guidelines [23], specific prompts were developed to facilitate automated screening using Python (version 3.9.0; Python Software Foundation). These prompts, structured to output results in a tabular format, instructed the LLM to perform screening based on the Population, Intervention, Control, Outcome, and Study design (PICOS) framework criteria defined for each topic study (detailed in Table S1 in Multimedia Appendix 1). An example prompt is provided in Figure S1 in Multimedia Appendix 1.

In addition, 2 LLMs, ChatGPT-4o (OpenAI) and Claude-3.5 sonnet (Anthropic), were used as independent reviewers to independently screen titles and abstracts. Study selection was based on the predefined inclusion and exclusion criteria. Discrepancies in study selection between the 2 LLMs were resolved by Gemini-1.5 pro (Google). Full-text papers underwent an identical screening process. The literature screening process assisted by LLM was conducted and supervised by 2 reviewers (Z-YD and F-QW). The detailed prompts used in LLM-assisted literature screening are provided in section S1 in Multimedia Appendix 2.

We then evaluated the performance of 2 open-source, machine learning–based screening tools, ASReview [24,25] and Abstrackr [26,27], for title and abstract screening. We compared their results against conventional manual screening methods, which served as the reference standard, to assess the accuracy of LLM-assisted literature screening. The detailed methodology is documented in section S2 in Multimedia Appendix 2.

### Statistical Analysis

The accuracy of both LLM-assisted literature screening and 2 semiautomated, machine learning–based screening tools were evaluated in each topical study using sensitivity, specificity, and their corresponding 95% CIs. The primary analysis focused on the sensitivity and specificity of LLM-assisted literature screening assessed after full-text review. The secondary analysis focused on the sensitivity and specificity of LLM-assisted screening at the title and abstract review stage. In addition, meta-analysis techniques were used to calculate pooled sensitivity and specificity, along with the summary receiver operating characteristic (SROC) curve. This provided overall results for the primary, secondary, and post hoc analyses. Heterogeneity across topic studies was assessed by calculating the inconsistency value (*I*²) using the chi-square test. A random-effects model was used to pool sensitivity and specificity if *I*² exceeded 50% or if the *P* value was less than .05. Meta-analyses were conducted using Stata (version 15.0; StataCorp), while other statistical analyses were performed using GraphPad Prism (version 8.0.1; GraphPad Prism, Inc).

A post hoc analysis (conducted by YW and QP) involved reviewing papers of false-negative classifications to identify the sources of LLM errors during screening (the comprehensive explanations for the occurrence of false-negative classifications are provided in Table S2 in Multimedia Appendix 1). Subsequently, the literature screening prompts were refined by incorporating a chain-of-thought prompting strategy [28] based on the identified error patterns (conducted by CS). In addition, 3 iterations of screening querying were then performed with revised prompts to optimize the model’s validity. A study was considered eligible if the LLM classified it as eligible during any of the 3 iterations.

To further assess the robustness of LLM-assisted screening and account for potential variations, a separate post hoc meta-analysis was conducted (Y-LJ and Z-YL). This analysis compared the pooled effect sizes derived from LLM-assisted screening (including only true positives) with those from conventional screening (including both true positives and false negatives) for each topic study.

### Ethical Considerations

The study did not involve human participants or biological specimens. As such, the Biomedical Ethics Committee of West China Hospital, Sichuan University determined that this research was eligible for exemption from ethical review (reference number: 2024−1177). This decision aligns with institutional and local policies that exempt studies not involving human subjects or biological materials from requiring formal ethics board approval.

## Results

### The Results of Conventional Manual and LLM-Assisted Literature Screening

**Figure 1:**
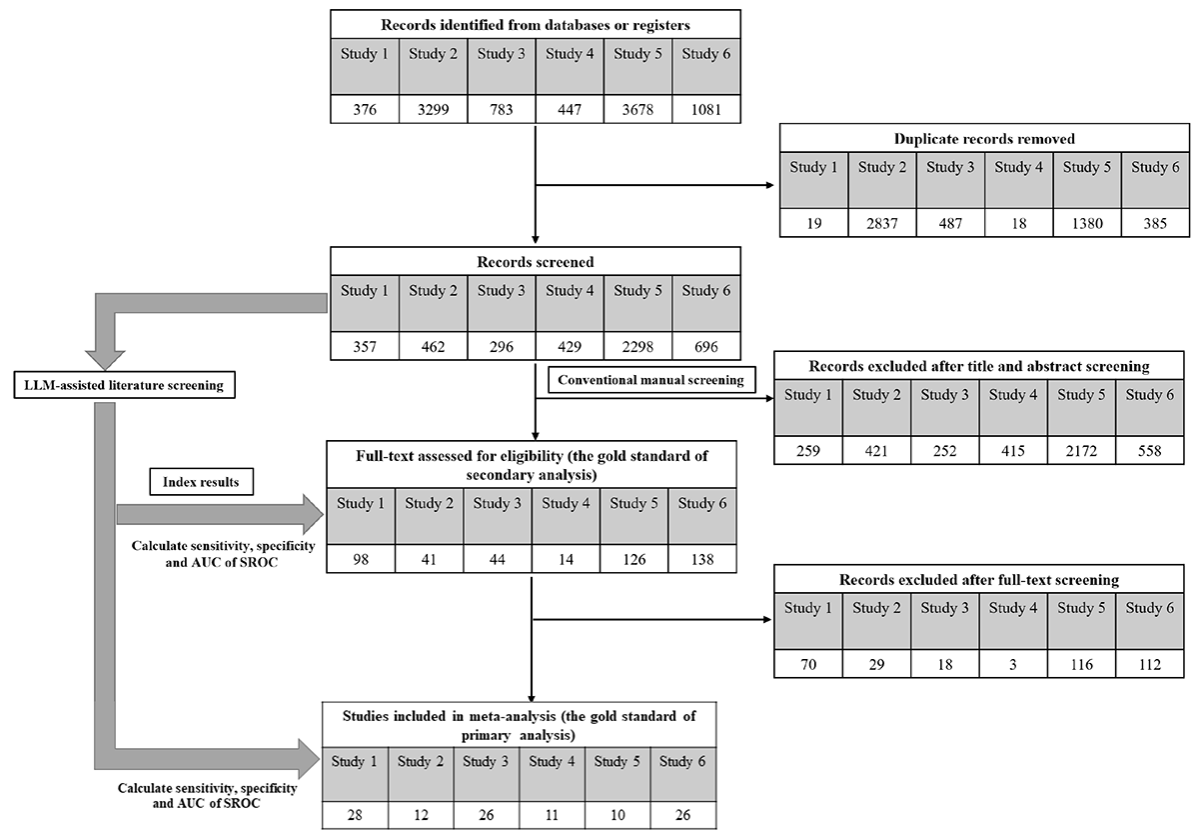
Flow diagram of meta-analysis using conventional manual literature screening and large language model (LLM)–assisted screening. AUC: area under the curve; SROC: summary receiver operating characteristic curve.

Following deduplication in the conventional literature screening process, the initial search yielded 357, 462, 296, 429, 2,298, and 696 papers for studies 1-6, respectively. Title and abstract screening resulted in 98, 41, 44, 14, 126, and 138 papers selected for full-text review in the corresponding studies. Ultimately, 28, 12, 26, 11, 10, and 26 papers from studies 1 to 6, respectively, met the inclusion criteria and were incorporated into the final meta-analysis (Figure 1). The results of LLM-assisted literature screening are listed and described in Table S3 in Multimedia Appendix 1 and section S3 in Multimedia Appendix 2.

### Primary Analysis of LLM-Assisted Literature Screening

**Figure 2:**
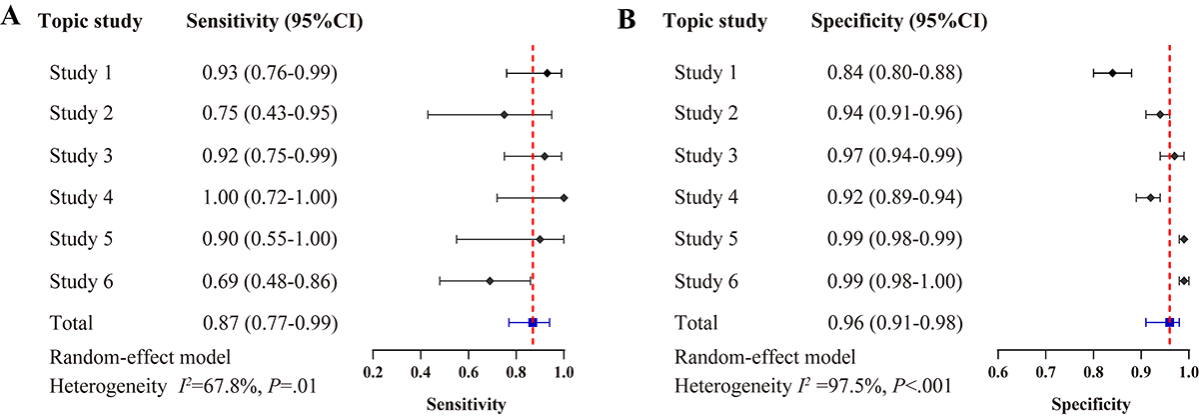
Sensitivity and specificity of large language model (LLM)–assisted literature screening in the primary analysis.

In the LLM-assisted literature screening process, a total of 26, 9, 24, 11, 9, and 18 papers from studies 1 to 6, respectively, were included in the final meta-analysis (Table S3 in Multimedia Appendix 1). The primary analysis revealed the sensitivity and specificity of the LLM-assisted screening for studies 1-6 as follows: 0.93 (95% CI 0.76-0.99) and 0.84 (95% CI 0.80-0.88), 0.75 (95% CI 0.43-0.95) and 0.94 (95% CI 0.91-0.96), 0.92 (95% CI 0.75-0.99) and 0.97 (95% CI 0.94-0.99), 1.00 (95% CI 0.72-1.00) and 0.92 (95% CI 0.89-0.94), 0.90 (95% CI 0.55-1.00) and 0.99 (95% CI 0.98-0.99), and 0.69 (95% CI 0.48-0.86) and 0.99 (95% CI 0.98-1.00), respectively (Figure 2). Meta-analysis of 6 topic studies revealed that LLM-assisted screening demonstrated a high discriminative ability, with SROC curve analysis yielding an area under the curve (AUC) of 0.96 (95% CI 0.94-0.97); see Figure S2 in Multimedia Appendix 1. Furthermore, the pooled sensitivity and specificity were 0.87 (95% CI 0.77-0.99) and 0.96 (95% CI 0.91-0.98), respectively (Figure 2). The counts of true positives, false negatives, false positives, and true negatives of primary analysis are detailed in Table S3 in Multimedia Appendix 1.

### Secondary Analysis of LLM-Assisted Screening

**Figure 3:**
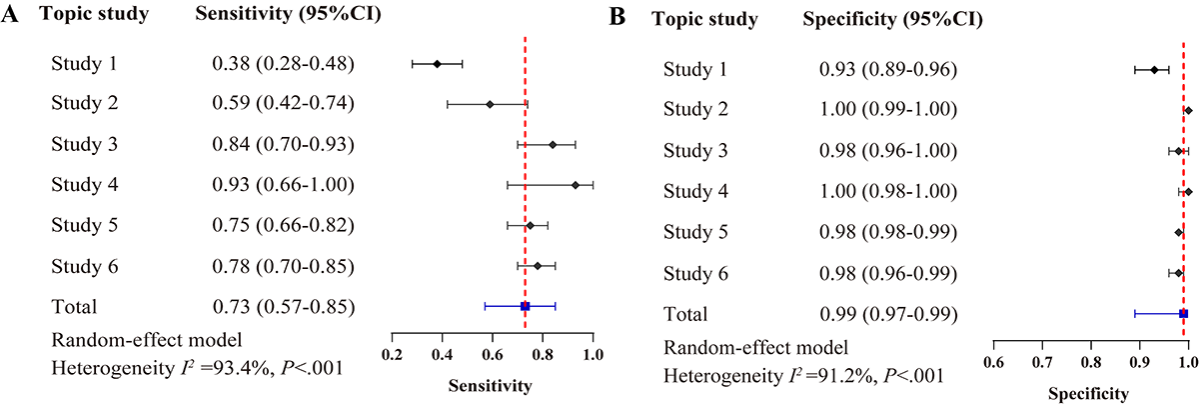
Sensitivity and specificity of large language model (LLM)–assisted literature screening in the secondary analysis.

The pooled sensitivity and specificity across these 6 studies were 0.73 (95% CI 0.57-0.85) and 0.99 (95% CI 0.97-0.99), respectively (Figure 3 and section S4 in Multimedia Appendix 2). The SROC analysis yielded an AUC of 0.97 (95% CI 0.96-0.99); see Figure S2 in Multimedia Appendix 1. The counts of true positives, false negatives, false positives, and true negatives of secondary analysis are detailed in Table S3 in Multimedia Appendix 1.

The pooled sensitivity and specificity of ASReview tool-assisted screening were 0.58 (95% CI 0.53-0.64) and 0.97 (95% CI 0.91-0.99), respectively (Figure S3 in Multimedia Appendix 1). The pooled sensitivity and specificity of Abstrackr tool-assisted screening were 0.48 (95% CI 0.35-0.62) and 0.96 (95% CI 0.88-0.99), respectively (Figure S4 in Multimedia Appendix 1). The SROC analysis yielded AUC values of 0.66 (95% CI 0.62-0.70) and 0.78 (95% CI 0.74-0.82), respectively (Figure S5 in Multimedia Appendix 1). The corresponding counts of true positives, false negatives, false positives, and true negatives of the title and abstract screening phase are detailed in Table S4 in Multimedia Appendix 1.

### Post Hoc Analysis of Revised Prompts

**Figure 4:**
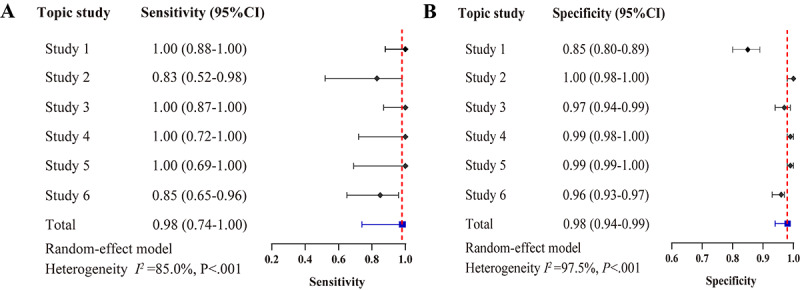
Sensitivity and specificity of large language model (LLM)–assisted literature screening: primary analysis with revised prompt (post hoc).

A post hoc analysis was conducted using a revised prompt (Figure S6 in Multimedia Appendix 1) and incorporating a chain-of-thought strategy (Table S2 in Multimedia Appendix 1 and section S5 in Multimedia Appendix 2). The sensitivity and specificity of LLM-assisted screening in the primary analysis are described in section S6 in Multimedia Appendix 2. The overall sensitivity and specificity across these 6 studies in the primary analysis were 0.98 (95% CI 0.74-1.00) and 0.98 (95% CI 0.94-0.99), respectively (Figure 4). The SROC analysis yielded an AUC of 1.00 (95% CI 0.99-1.00; Figure S7 in Multimedia Appendix 1). The counts of true positives, false negatives, false positives, and true negatives for the post hoc analysis are detailed in Table S3 in Multimedia Appendix 1. The sensitivity, specificity, and AUC of LLM-assisted screening in the secondary analysis are presented and described in Figures S7-S8 in Multimedia Appendix 1 and section S7 in Multimedia Appendix 2.

### Post Hoc Meta-Analysis of Index Results of LLM-Assisted Screening

This post hoc meta-analysis compared pooled effect sizes from studies 1, 2, 3, 5, and 6 (Table S5 in Multimedia Appendix 1 and section S8 in Multimedia Appendix 2) for LLM-assisted screening versus conventional screening. The meta-analysis revealed comparable results between the two methods. Furthermore, the false-negative papers did not substantially affect the overall conclusions of the corresponding topic studies (Figures S9-S17 in Multimedia Appendix 1).

## Discussion

### Principal Findings

Our study addresses a critical challenge in the development of clinical practice guidelines—the labor-intensive and time-consuming nature of literature screening in meta-analyses [2]. Traditionally, this process relies heavily on manual efforts to ensure the inclusion of high-quality evidence from randomized controlled trials and cohort studies [2,3,8]. While machine learning approaches have been explored, they often lack the precision required for reliable screening [8]. In this context, our research highlights the potential of LLMs to enhance the efficiency and accuracy of literature screening. Our findings suggest that LLMs, with their advanced natural language processing capabilities, can effectively automate significant portions of the screening process, aligning closely with the accuracy of manual methods. This advancement could significantly streamline the preparation of systematic reviews and meta-analyses, offering a promising alternative to traditional methods and addressing the limitations observed with earlier machine learning models.

Our primary analysis revealed that using literature included after conventional manual full-text review as the reference standard, the sensitivity and specificity of LLM-assisted literature screening ranged from 0.77 to 0.99 and 0.91 to 0.98, respectively. Furthermore, the SROC curve, constructed based on the true positive and false positive results from 6 studies, indicated a high level of accuracy for LLM-assisted literature screening, with an AUC ranging from 0.94 to 0.97. Post hoc analysis incorporating modified prompts demonstrated that LLM-assisted literature screening achieved even higher sensitivity (0.98, 95% CI 0.74-1.00) while maintaining a similarly high level of specificity and AUC (0.94-0.99 and 0.99-1.00, respectively). Currently, limited research has explored the accuracy of LLM-assisted screening in meta-analysis for the development of high-level evidence-based medicine. Our research establishes a foundation for the future application of LLMs in the literature screening process of meta-analyses.

### Comparison With Previous Work

Our study results demonstrate that LLM-assisted literature screening offers superior accuracy compared with traditional machine-learning models. Previous studies [24,27,29] have reported relatively low sensitivity for machine learning–assisted literature screening, ranging from 0.24 to 0.80. Our study also found that 2 semiautomated tools used for title and abstract screening in literature reviews had relatively low sensitivity, ranging from 0.35 to 0.64. The advantages of LLMs are evident in 3 key aspects. First, large language models possess advanced capabilities in language understanding and text generation, surpassing the capabilities of traditional screening tools [15]. This distinctive feature enables LLMs to excel at identifying relevant literature and discerning irrelevant studies. Conversely, machine learning models require predefined training and validation datasets, including key literature inputs, and often necessitate human review, thereby increasing the barrier to their implementation [24,27]. In contrast, LLMs can rapidly and efficiently generate screening results without the need for training data or human review, owing to their user-friendly conversational interface. This significantly enhances efficiency and reduces workload. Furthermore, research suggests that LLM-assisted screening can achieve a tenfold reduction in screening time compared with manual screening [1]. While machine learning models also offer time-saving benefits, LLMs eliminate the need for training data and key literature inputs, potentially yielding even greater time savings. Second, previous studies [14,29-31] using machine learning and other natural language processing tools for literature screening reported sensitivities ranging from 0.75 to 0.90, which is consistent with the sensitivity observed in our study. However, our findings indicate that the specificity of LLM-assisted screening (95% CI 0.91-0.98) was notably higher than that reported in previous studies (95% CI 0.69-0.90), suggesting a potential advantage of LLMs in accurately identifying literature relevant to the research topic. While LLMs exhibited very high specificity in both primary, second, and post hoc analyses, it is important to acknowledge that these high-performance estimates might be partially attributed to an overrepresentation of true-negative literature in the datasets used. Third, LLMs exhibit a capacity for continuous learning and self-improvement, akin to human learning processes. With appropriate prompts and instructions, LLMs can refine their performance iteratively, leading to progressively enhanced accuracy. New iterations of LLMs are released approximately every 3-6 months, and these updates are anticipated to further improve sensitivity and specificity during literature screening in meta-analyses. Furthermore, LLMs offer broad applicability and functional extensibility across diverse topics and formats, enabling users to develop customized chatbots tailored to specific research needs. These advantages collectively lower the barrier to entry, reduce workload, and maintain high levels of accuracy, potentially revolutionizing the literature screening process in the future.

In contrast to previous studies [1,9,14] that used a single LLM for literature screening, this study used 3 models concurrently, thereby more accurately reflecting the conventional manual screening process. For instance, Oami et al [1]. relied solely on the ChatGPT-4 Turbo model (released November 7, 2023). Recognizing the ongoing evolution of LLMs, this study expanded the model set to include the updated ChatGPT-4o (released May 13, 2024), Claude-3.5 Sonnet (released June 21, 2024), and Gemini-1.5 Pro (released May 14, 2024). The combined sensitivity and specificity of LLM-assisted literature screening achieved in this study were 0.87 (95% CI 0.77-0.99) and 0.96 (95% CI 0.91-0.98), respectively. These results surpass the sensitivity of 0.75 (95% CI 0.43-0.92) reported by Oami et al [1], suggesting that updated LLMs may enhance screening sensitivity. Specificity remained consistent with the 0.99 (95% CI 0.99-0.99) reported by Oami et al [1], highlighting the potential of LLMs to effectively identify irrelevant literature. While a direct comparison may be limited due to potential heterogeneity introduced by differing research themes, this study provides a novel approach and establishes a foundation for the broader application of LLMs in facilitating literature screening for meta-analyses.

Post hoc analysis revealed that modified prompts significantly improved the sensitivity and specificity of LLM-assisted literature screening to 0.98 (95% CI 0.74-1.00) and 0.98 (95% CI 0.94-0.99), respectively. This underscores the substantial impact of prompt content on LLM performance in literature screening and the quality of meta-analyses. Recent research on prompt engineering has demonstrated the influence of prompts on LLM performance and proposed strategies for tailoring LLM responses to specific topics [17,32,33]. In this study, prompts were designed based on the PICOS framework for each research topic. During the post hoc analysis, false-negative results from studies 1, 2, 3, 5, and 6 were reviewed. This analysis indicated a positive correlation between the complexity of inclusion criteria and the likelihood of LLMs making “exclude” decisions. LLMs exhibited a tendency to strictly adhere to the inclusion criteria specified in the prompts. Conversely, human reviewers typically apply inclusion criteria more conservatively during the initial title and abstract screening phase to minimize the risk of overlooking potentially relevant studies. Based on this observation, it was determined that minor discrepancies between the inclusion criteria in the prompts and the titles or abstracts could be tolerated. Consequently, the prompts were revised to relax the inclusion criteria. In total, 3 iterations of inquiries were then conducted with the modified prompts to optimize sensitivity and reduce false-negative results. The post hoc analysis confirmed an improvement in the sensitivity of LLM-assisted literature screening. However, some false-negative literature was still missed in the final comprehensive analysis, potentially impacting the robustness of the meta-analysis conclusions.

To assess the impact of false-negative literature on the final conclusions, a separate post hoc meta-analysis was conducted. This analysis compared the pooled effect sizes derived from LLM-assisted screening (including only true positives) with those derived from conventional screening (including both true positives and false negatives) for topic studies. The results indicated comparable outcomes between the two methods in most topic studies. Furthermore, the false-negative papers did not substantially alter the overall conclusions of the corresponding topic studies in the majority of instances. Notably, however, for outcomes on lymph node dissection (study 3) and overall survival (study 5), the exclusion of false negatives shifted the results from statistically significant positive effects to nonsignificant negative effects. An examination of the studies included in the study 3 and 5, focusing on the outcomes of lymph node dissection and overall survival, revealed significant heterogeneity and publication bias in the reported findings. This suggests that in meta-analyses demonstrating low heterogeneity and an absence of publication bias, the inclusion or exclusion of potential false-negative studies may not substantially impact the overall conclusions. However, these findings underscore the need for further research on the application of LLMs for literature screening in meta-analyses. Future research should focus on developing prompts that are more readily interpretable by LLMs and exploring techniques for continuous self-correction within LLMs to improve sensitivity.

### Limitations

This study acknowledges several limitations. First, its focus is exclusively on meta-analyses within thoracic surgery. Therefore, the generalizability and broader applicability of LLM-assisted literature screening to other fields require further investigation. Future research should evaluate the performance of LLMs across diverse meta-analysis fields to assess their validity for literature screening across various domains of evidence-based medicine. Second, ongoing updates to LLMs may introduce variations in the quality of model outputs over time, potentially influencing the strength of evidence synthesized in meta-analyses. Third, the use of conventional manual screening as a reference standard may introduce inherent errors in inclusion and exclusion decisions. Existing research [34] indicates that error rates for human reviewers in literature screening range from 6.68% to 21.11% across different fields, with an average error rate of 10.76%. This suggests that the reference standard itself is not infallible, and a comparable error rate for LLM-assisted screening could be considered acceptable. Fourth, a key limitation of our study is the use of previously published systematic reviews, which raises the risk of bias, as the content of these reviews may have been included in the training materials for LLMs. To mitigate this, we redesigned the search strategy and replicated the inclusion criteria and timeframes outlined in these meta-analyses for a new round of literature retrieval and screening. In addition, we evaluated the results with new readers to ensure the integrity of our findings. Fifth, an additional key limitation of our study lies in the method of accessing LLMs, as we used a web-based interface instead of an application programming interface (API). While convenient, web-based access lacks the flexibility, performance, offline functionality, and data security provided by API-based deployment. This reliance on external servers outside the researchers’ control may have introduced some bias in the literature screening process. Future studies should explore API-based access, which enables local or server deployment, offering greater control, enhanced security, and better integration with research workflows. APIs also allow secure handling of sensitive data and more efficient operation in offline or resource-constrained settings. Although the web-based interface was sufficient for this study, adopting API-based access in future research could address these limitations and improve reliability and security.

Despite these limitations, the integration of LLMs into the meta-analysis workflow represents a significant advancement with the potential to enhance productivity and accelerate the speed and quality of resource and knowledge synthesis.

### Conclusions

LLM-assisted screening, particularly at the full-text screening level and with revised prompts, can achieve accuracy comparable with manual screening. This suggests LLMs hold significant potential for streamlining literature screening in systematic reviews, reducing workload without sacrificing quality. Integrating LLMs into evidence synthesis workflows could accelerate the production of high-quality reviews, facilitating more timely translation of research into practice and policy.

## Appendix

Multimedia Appendix 1 Additional tables and figures.

Multimedia Appendix 2 Additional material content.

Multimedia Appendix 3 CONSORT-EHEALTH Checklist.

## Abbreviations

API: application programming interface
AUC: area under the curve
CONSORT-EHEALTH: Consolidated Standards of Reporting Trials of Electronic and Mobile Health Applications and Online Telehealth
LLM: large language model
PICOS: Population, Intervention, Control, Outcome, and Study
SROC: summary receiver operating characteristic curve
STARD: Standards for Reporting of Diagnostic Accuracy Studies

## Data Availability

All data generated or analyzed during this study are included in this published article and Multimedia Appendices 1 and 2.
