# Supplementary material for: Accuracy of Large Language Models for Literature Screening in Thoracic Surgery: Diagnostic Study
Source: J Med Internet Res. 2025 Mar 11;27:e67488. doi: 10.2196/67488 (PMC11937709; doi:10.2196/67488)
Supplement: Multimedia Appendix 1 [file jmir_v27i1e67488_app1.docx]

**Appendix-A**

**Table S1.** The Inclusion and Exclusion Criteria of Each Topic Study.

**Table S2.** List of Unidentified Articles (false-negative) in Large Language Model-Assisted Literature Screening for the Final Meta-Analysis of Each Topic Study.

**Table S3.** The Results of Large Language Model-Assisted Literature Screening.

**Table S4.** The Results of Machine Learning - Assisted Literature Screening.

**Table S5.** Pooled Effect Sizes of LLM-assisted (false-negative literatures excluded) and Conventional Literature Screening (full literatures included).

**Figure S1.** Original Prompt of the LLM-assisted Literature Screening Task.

**Figure S2.** The Summary Receiver Operating Characteristics Curve of LLM-assisted Literature Screening in the Primary and Secondary Analysis Using Original Prompt.

**Figure S3.** The Individual and Pooled Sensitivity and Specificity of ASRview Tool-assisted Literature Screening in Title and Abstract Screening Phase.

**Figure S4.** The Individual and Pooled Sensitivity and Specificity of Abstrackr Tool-assisted Literature Screening in Title and Abstract Screening Phase.

**Figure S5.** The Summary Receiver Operating Characteristics Curve of ASReview and Abstrackr tool-assisted Literature Screening

**Figure S6.** Revised Prompt Incorporating the Chain-of-though strategy for the LLM-assisted Literature Screening Task in Post Hoc Analysis.

**Figure S7.** The Summary Receiver Operating Characteristics Curve of LLM-assisted Literature Screening for the Primary and Secondary Analysis in the Post Hoc Analysis using revised prompt

**Figure S8.** Sensitivity and Specificity of LLM-assisted Literature Screening: Secondary Analysis with Revised Prompt (Post Hoc).

**Figure S9.** Forest Plots of LLM-assisted Screening vs. Conventional Manual Screening for Overall Survival in Stage IA Non-Small Cell Lung Cancer in Topic Study 1.

**Figure S10.** Forest Plots of LLM-assisted Screening vs. Conventional Manual Screening for Overall Survival in Stage IA$<$2cm Non-Small Cell Lung Cancer in Topic Study 1.

**Figure S11.** Forest Plots of LLM-assisted Screening vs. Conventional Manual Screening for Recurrence-Free Survival in Stage IA$<$2cm Non-Small Cell Lung Cancer in Topic Study 1.

**Figure S12.** Forest Plots of LLM-assisted Screening vs. Conventional Manual Screening for Overall Survival in Topic Study 2.

**Figure S13.** Forest Plots of LLM-assisted Screening vs. Conventional Manual Screening for Postoperative Hospital Time in Topic Study 3.

**Figure S14.** Forest Plots of LLM-assisted Screening vs. Conventional Manual Screening for Lymph Node Dissection in Topic Study 3.

**Figure S15.** Forest Plots of LLM-assisted Screening vs. Conventional Manual Screening for Overall Survival in Topic Study 5.

**Figure S16.** Forest Plots of LLM-assisted Screening vs. Conventional Manual Screening for Overall Survival in Topic Study 6.

**Figure S17.** Forest Plots of LLM-assisted Screening vs. Conventional Manual Screening for Recurrence-Free Survival in Topic Study 6.

**Table S1. The Inclusion and Exclusion Criteria of Each Topic Study**

| **Topic study** | **Title** | **Inclusion criteria** | | | | | **Exclusion criteria** |
| --- | --- | --- | --- | --- | --- | --- | --- |
|  |  | Population | Intervention | Control | Outcomes | Others (study design) |  |
| Study 1 | Segmentectomy or lobectomy for early-stage non-small-cell  lung cancer: a systematic review and meta-analysis | stage I lung cancer patients. | segmentectomy | lobectomy | The study investigates at least one of the following outcomes: OS, CSS or RFS.  The outcomes are reported for stage I, stage IA or stage IA <2cm. | The article contains enough information to calculate the hazard ratio (HR) with its associated standard error. | The study designs were reviews, case reports, letters to the editor and articles published in a language other than English; When the same patient data were analyzed in different studies, only the most recent one was included |
| Study 2 | Systematic review and meta-analysis of  video-assisted thoracoscopic surgery  segmentectomy versus lobectomy for stage  I non–small cell lung cancer | only included patients with clinical stage I and/or IA and/or IB NSCLC | video-assisted thoracoscopic surgery  segmentectomy | video-assisted thoracoscopic surgery  lobectomy | compared the perioperative and/or survival outcomes (follow-up time≥5years). | Publication in English; When the same data or data subsets were reported in more than one study, the one with the most details or the one most recently published was chosen. | Case-only designs, case reports, systematic reviews, meta-analyses, and animal studies |
| Study 3 | Updated Evaluation of Robotic- and Video-Assisted Thoracoscopic Lobectomy or Segmentectomy for Lung Cancer: A Systematic Review and Meta-Analysis | all patients had lung cancer defined histologically | robotic-assisted thoracoscopic surgery (RATS) | video-assisted thoracoscopic surgery (VATS) | at least one outcome was reported in the literature, including operation time, intraoperative bleeding, tumor size, R0 rate, conversion rate, lymph node harvested, and spleen preservation rate | randomized controlled trials (RCTs) or propensity score matching (PSM) studies, or retrospective studies, or cohort studies, or case–control studies comparing RATS and VATS; if repeated studies were published from the same center, captured the latest data and PSM data for analysis; language restrictions: English. | conference, abstracts, editorials, letters, and case reports and no comparative analysis between RATS and VATS. |
| Study 4 | Oncological Outcomes of Segmentectomy versus Lobectomy in  Clinical Stage I Non-Small Cell Lung Cancer up to Two  Centimeters: Systematic Review and Meta-Analysis | Studies including subjects with NSCLC | segmentectomy | lobectomy | Studies reporting overall survival (OS); Outcomes correlated to stage I with tumor with diameter ≤2cm | Randomized controlled trial, prospective or retrospective study designs | Studies including wedge resections; When the same patient data were analyzed in different studies, only the most recent one was included; Reviews; Case reports; Letters; Language other than English. |
| Study 5 | Lobectomy versus segmentectomy for stage IA3 (T1cN0M0) non-small cell lung cancer: a meta-analysis and systematic review | patients diagnosed with stage IA3 (T1cN0M0) NSCLC based on The Eighth Edition Lung Cancer Stage Classification | segmentectomy | lobectomy | Outcomes: survival, intraoperative outcomes, hospitalization outcomes, recurrences, and complications | Study design: randomized controlled trial (RCT) or cohort study; When the same patient populations were involved in 2 or more studies, RCT and propensity score matching study would be prioritized, and if not, study with the largest sample size should be prioritized for inclusion. | Animal experiments, meta-analyses, letters, commentaries, and reviews |
| Study 6 | Differential efficacy of segmentectomy and wedge resection in sublobar resection compared to  lobectomy for solid-dominant stage IA lung cancer:  a systematic review and meta-analysis | solid-dominant (consolidation-to-tumor ratio (CTR) ≥50%) clinical stage IA lung  cancer | sublobar resection (including segmentectomy and wedge resec  tion) | lobectomy | reporting at  least one outcome of interest, including OS or RFS | randomized controlled trial  (RCT) and retrospective cohort studies (RCS); publication in English language. | studies with overlapping study populations; review articles, editorials, letters, abstracts, non-original studies, and animal studies, and studies that did not report the outcomes of interest. |

NSCLC, non-small cell lung cancer; OS, overall survival; CSS, cancer-specific survival; RFS, recurrence-free survival.

**Table S2.** **List of Unidentified Articles (false-negative) in Large Language Model-Assisted Literature Screening for the Final Meta-Analysis of Each Topic Study**

| **Topic Study** | **First-author** | **Year** | **Title** | **Reason of exclusion** |
| --- | --- | --- | --- | --- |
| Study 1 | Hattori^a^ | 2017 | Locoregional recurrence after segmentectomy for clinical-T1aN0M0 radiologically solid non-small-cell lung carcinoma | The focus of the study is on locoregional recurrence in segmentectomy for clinical T1aN0M0 NSCLC, with no direct comparison to lobectomy. The meta-analysis (Study 1) investigates at least one of the following outcomes: overall survival, cancer-specific survival, and recurrence-free survival, rather than locoregional recurrence. Additionally, the inclusion criteria for Study 1 compare lobectomy to segmentectomy |
| Study 1 | Campione^a^ | 2004 | Comparison between segmentectomy and larger resection of stage IA non-small cell lung carcinoma | Insufficient details on outcomes (overall survival, cancer-specific survival and recurrence-free survival) |
| Study 2 | Echavarria^a^ | 2016 | Comparison of pulmonary function tests and perioperative outcomes after robotic-assisted pulmonary lobectomy vs segmentectomy | The key determinant for exclusion is the focus on robotic-assisted procedures rather than standard VATS procedures and the lack of details about long-term survival outcomes and follow-up duration required by the inclusion criteria. Even though there is some uncertainty (mainly about clinical staging), the mismatch in the type of surgical procedure and lack of required survival data strongly indicate exclusion. |
| Study 2 | Roman | 2019 | Lobectomy vs. segmentectomy. A propensity score matched comparison of outcomes | Despite fulfilling several inclusion criteria, the inclusion of patients with stage II/IIIa NSCLC does not align with the focus on stage I, IA, or IB specified by the systematic review criteria. Since the patient population is a key aspect of the eligibility criteria, the study should be excluded to maintain consistency and relevance in the review's focus on early-stage lung cancer |
| Study 2 | Soukiasian | 2012 | Video-assisted thoracoscopic trisegmentectomy and left upper lobectomy provide equivalent survivals for stage IA and IB lung cancer | The study compares video-assisted thoracoscopic surgery (VATS) trisegmentectomy to VATS lobectomy. While trisegmentectomy is a form of sublobar resection, it focuses specifically on left upper lobe (LUL) trisegmentectomies, which may not universally represent segmentectomy in the context required by the review. By focusing specifically on LUL trisegmentectomy, the study provides a more specialized comparison that might not reflect broader segmentectomy versus lobectomy outcomes across all lung sections. Given the need for broadly applicable insights in the systematic review, this more specialized procedure does not fully align with the typical segmentectomy being considered, thus warranting exclusion. |
| Study 3 | Chen^a^ | 2021 | Cost-effectiveness evaluation of robotic-assisted thoracoscopic surgery versus open thoracotomy and video-assisted thoracoscopic surgery for operable non-small cell lung cancer | Exclusion criteria:  The study primarily focuses on cost-effectiveness, not on the direct comparison of clinical outcomes between RATS and VATS, which is a requirement. Cost-effectiveness studies are not included as they do not primarily examine clinical outcomes directly between the specified surgical types. Therefore, based on the exclusion criteria related to the type of analysis and the focus on cost rather than direct comparative outcomes, this study should be excluded from your review |
| Study 3 | Deen^a^ | 2014 | Defining the cost of care for lobectomy and segmentectomy: a comparison of open, video-assisted thoracoscopic, and robotic approaches | The study involves patients undergoing lung resection for early-stage lung cancer, carcinoid, or metastatic foci, which aligns with the lung cancer focus, but it does not specify if lung cancer is histologically confirmed for all patients in the context of the criteria. The study compares open surgery, robotic-assisted thoracoscopic surgery (RATS), and video-assisted thoracoscopic surgery (VATS). However, your criteria specify the need to compare RATS and VATS specifically, excluding open thoracotomy (OT) from the primary intervention comparison. The focus on cost without the specified clinical outcomes and the inclusion of open surgery in the comparative analysis are key reasons for exclusion. Therefore, this study should not be included in your systematic review and meta-analysis based on the criteria provided. |
| Study 5 | Peng^a^ | 2022 | Lobectomy offers improved survival outcomes relative to segmentectomy for >2 but ≤4 cm non-small cell lung cancer tumors | The study focuses on patients with NSCLC tumors >2 but ≤4 cm. The eligible population for the systematic review is patients diagnosed with stage IA3 (T1cN0M0) NSCLC, which specifically pertains to tumors >2 cm but ≤3 cm as defined by The Eighth Edition Lung Cancer Stage Classification. The study includes tumors >3 cm ≤ 4 cm, which does not fit the criteria for stage IA3. |
| Study 6 | Inoue | 2010 | Clinical outcome of resected solid-type small-sized c-stage IA non-small cell lung cancer | Focuses on older patient demographics and surgical techniques without specific outcomes such as OS or RFS. It does not directly compare sublobar resection and lobectomy. It seems eligibility criteria were not met. |
| Study 6 | Jeon^a^ | 2014 | Sublobar resection versus lobectomy in solid-type, clinical stage IA, non-small cell lung cancer | Primarily focuses on propensity score matching in non-surgical treatments and lacks direct comparison of segmentectomy and lobectomy. The emphasis is on statistical methodology without direct surgical comparison, which limits its relevance |
| Study 6 | Kamigaichi^a^ | 2020 | Prognosis of segmentectomy and lobectomy for radiologically aggressive small-sized lung cancer | Specifically focuses on an RCT conducted within a single geographic region without providing generalizable data. It does not consistently report on specified outcomes like OS or RFS across various demographics, thus limiting the scope and applicability. |
| Study 6 | Mimae^a^ | 2020 | Wedge resection as an alternative treatment for octogenarian and older patients with early-stage non-small-cell lung cancer | Discusses primarily lymph node dissection techniques and wedge resection procedures without making direct comparative analysis of segmentectomy and lobectomy based on outcomes like OS or RFS. The focus is too narrow on a procedure that doesn't meet the inclusion criteria. |
| Study 6 | Phillips | 2021 | Impact of Nodule Density in Women With Sublobar Resection for Stage IA Adenocarcinoma | The study is non-comparative and focuses on diagnostic techniques such as nodule density in women. It does not address the surgical outcomes like OS or RFS essential for this review, limiting its relevance to the criteria established. |
| Study 6 | Soh^a^ | 2022 | Limited resection for stage IA radiologically invasive lung cancer: a real-world nationwide database study | Main focus is on radiological evaluations and limited resection without specific surgical outcome comparisons. It does not provide a direct comparison of sublobar resection and lobectomy with relevant outcomes such as OS or RFS. |
| Study 6 | Suh | 2018 | Prognostic prediction of clinical stage IA lung cancer presenting as a pure solid nodule | Focus is on clinical stage IA staging accuracy rather than directly comparing sublobar versus lobectomy procedures. It does not address critical outcomes like OS or RFS, limiting its relevance. |
| Study 6 | Sun | 2020 | Solid component ratio influences prognosis of GGO-featured IA stage invasive lung adenocarcinoma | Analyzes solid tumor ratios but does not provide a direct surgical outcome comparison between sublobar resection and lobectomy. Its focus remains on radiological prognostic factors rather than procedural outcomes. |

^a^The article was included in the LLM-assisted literature screening using modified prompt for the *post hoc* analysis; NSCLC, non-small cell lung cancer; RATS, robot-assisted thoracoscopic surgery; VATS, video-assisted thoracoscopic surgery; OS, overall survival; RFS, recurrence-free survival; GGO, ground-glass opacity.

**Table S3. The Results of Large Language Model-Assisted Literature Screening**

| **After title/abstract screening** | **LLM-Using original prompt** | | | | **LLM-Using modified prompt** | | | |
| --- | --- | --- | --- | --- | --- | --- | --- | --- |
|  | **TP** | **FN** | **FP** | **TN** | **TP** | **FN** | **FP** | **TN** |
| Study 1 | 37 | 61 | 18 | 241 | 74 | 24 | 7 | 252 |
| Study 2 | 24 | 17 | 1 | 420 | 33 | 8 | 24 | 397 |
| Study 3 | 37 | 7 | 4 | 248 | 40 | 4 | 15 | 237 |
| Study 4 | 13 | 1 | 2 | 410 | 14 | 0 | 8 | 407 |
| Study 5 | 94 | 32 | 35 | 2137 | 108 | 18 | 26 | 2146 |
| Study 6 | 108 | 30 | 12 | 546 | 129 | 9 | 44 | 514 |
| **After full-text screening** | **TP** | **FN** | **FP** | **TN** | **TP** | **FN** | **FP** | **TN** |
| Study 1 | 26 | 2 | 51 | 278 | 28 | 0 | 38 | 221 |
| Study 2 | 9 | 3 | 29 | 421 | 10 | 2 | 2 | 419 |
| Study 3 | 24 | 2 | 8 | 262 | 26 | 0 | 8 | 240 |
| Study 4 | 11 | 0 | 34 | 384 | 11 | 0 | 3 | 409 |
| Study 5 | 9 | 1 | 27 | 2145 | 10 | 0 | 17 | 2155 |
| Study 6 | 18 | 8 | 5 | 665 | 22 | 4 | 25 | 533 |

LLM, large language model; TP, true positive; FN, false negative; FP, false positive; TN, true negative

**Table S4. The Results of Machine Learning - Assisted Literature Screening**

| **After title/abstract screening** | **ASReview^a^** | | | | **Abstrackr^a^** | | | |
| --- | --- | --- | --- | --- | --- | --- | --- | --- |
|  | **TP** | **FN** | **FP** | **TN** | **TP** | **FN** | **FP** | **TN** |
| Study 1 | 47 | 51 | 119 | 140 | 22 | 76 | 70 | 189 |
| Study 2 | 19 | 22 | 2 | 419 | 13 | 28 | 1 | 420 |
| Study 3 | 29 | 15 | 6 | 246 | 28 | 16 | 11 | 241 |
| Study 4 | 11 | 3 | 5 | 407 | 9 | 5 | 11 | 401 |
| Study 5 | 74 | 52 | 28 | 2144 | 70 | 56 | 101 | 2071 |
| Study 6 | 85 | 53 | 10 | 548 | 81 | 57 | 39 | 519 |

^a^ASReview and Abstrackr are two open-source semiautomated machine learning literature screening tools. TP, true positive; FN, false negative; FP, false positive; TN, true negative

**Table S5. Pooled effect sizes of LLM-assisted (false-negative literatures excluded) and conventional literature screening (full literatures included).**

| **Topic study** | **False-negative literature (first-author and year)** | **Outcome** | **Effect-size with 95%CI** | **LLM-assisted screening** | **Conventional manual screening** | **Whether the conclusion has changed** |
| --- | --- | --- | --- | --- | --- | --- |
| Study 1 | Campione 2004 | OS in stage IA NSCLC | HR | 0.96 (0.64,1.28) | 0.99 (0.69,1.30) | No |
| Study 1 | Hattori 2017 | OS in stage IA < 2cm NSCLC | HR | 0.93 (0.60,1.25) | 0.94 (0.63,1.25) | No |
| Study 1 | Hattori 2017 | RFS in stage IA < 2cm NSCLC | HR | 1.03 (0.70,1.36) | 1.06 (0.73,1.38) | No |
| Study 2 | Roman 2019  Soukiasian 2012 | OS | HR | 1.14 (0.84,1.43) | 1.01 (0.76,1.26) | No |
| Study 3 | Chen 2021 | Lymph node dissection | SMD | 0.26 (-0.01,0.54) | 0.28 (0.02,0.54) | Yes, but the difference in SMD values was small |
| Study 3 | Chen 2021  Deen 2014 | Postoperative hospital time | OR | 0.96 (0.89,1.03) | 0.95 (0.88,1.02) | No |
| Study 5 | Peng 2022 | OS | HR | 1.08 (0.85,1.31) | 1.15 (1.02,1.28) | Yes, but the difference in HR values was small |
| Study 6 | Inoue 2010  Jeon 2014  Kamigaichi 2020  Mimae 2020  Phillips 2020  Soh 2020 | OS | HR | 1.16 (0.76,1.55) | 1.13 (0.85,1.41) | No |
| Study 6 | Inoue 2010  Jeon 2014  Kamigaichi 2020  Mimae 2020  Phillips 2020  Soh 2020  Sun 2022  Suh 2018 | RFS | HR | 1.33 (0.99,1.68) | 1.22 (0.98,1.46) | No |

OS, overall survival; RFS, recurrence-free survival; HR, hazard ratio; SMD, standard man difference; OR, odds ratio; CI, confidence interval.

**
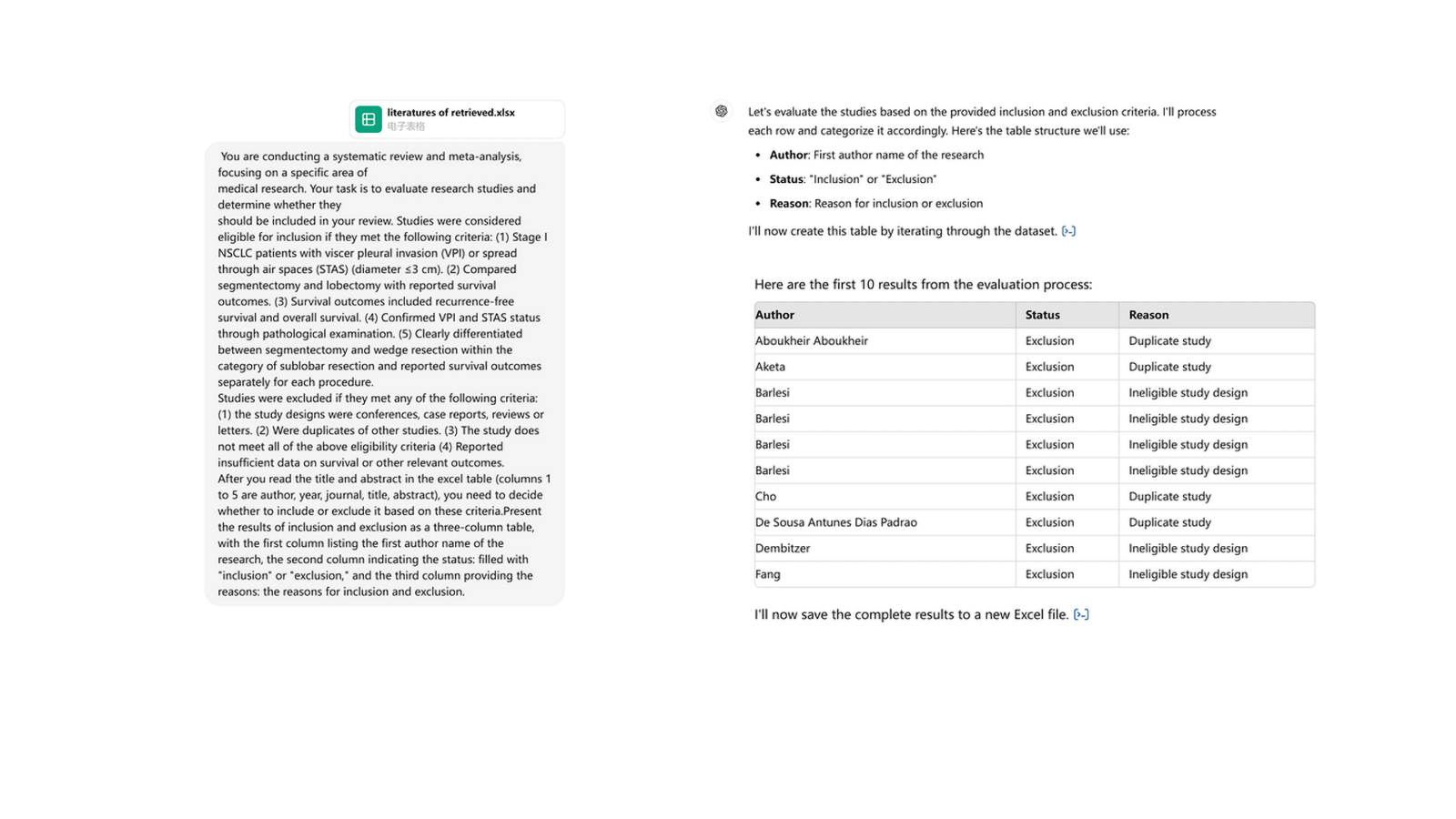
Figure S1. Original Prompt of the LLM-assisted Literature Screening Task.**

The prompt, structured to output results in a tabular format, instructed the LLM to perform screening based on the Population, Intervention, Control, Outcome, and Study design (PICOS) framework criteria defined for each topic study ( Table S1). This figure is an example prompt for literature screening in study 1.

**
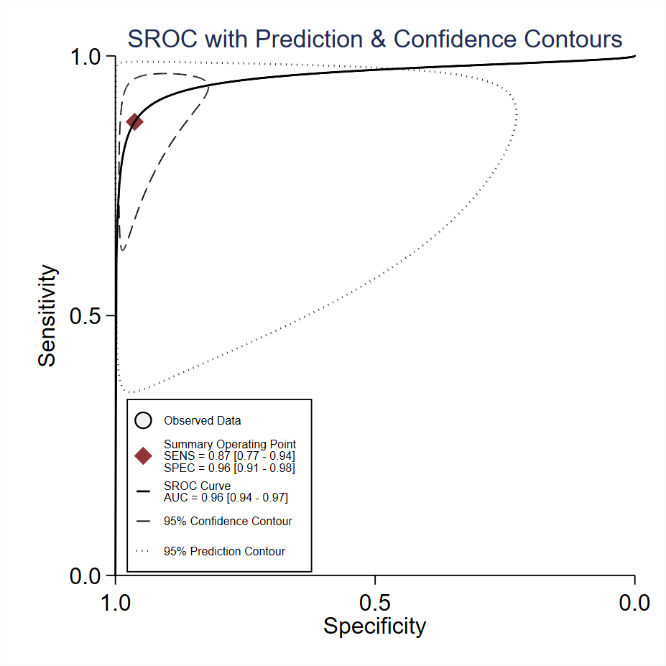

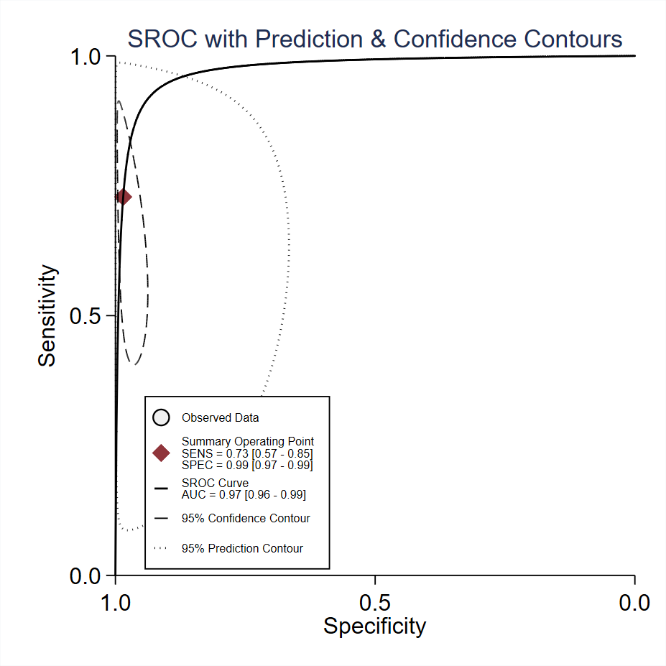
Figure S2. The Summary Receiver Operating Characteristics Curve of LLM-assisted Literature Screening in the Primary and Secondary Analysis Using Original Prompt.**

**A**

**B**

In the primary analysis using the original prompt (A), the Summary Receiver Operating Characteristics Curve (SROC) yielded an area under the curve (AUC) value of 0.96 (95%CI: 0.94-0.97). In the secondary analysis using original prompt (B), the SROC yielded an AUC value of 0.97 (95%CI: 0.96-0.99). Both the primary and secondary analysis revealed that LLM-assisted literature screening demonstrated a high discriminative ability.

**Figure S3. The Individual and Pooled Sensitivity and Specificity of ASRview Tool-assisted Literature Screening in Title and Abstract Screening Phase.**

**
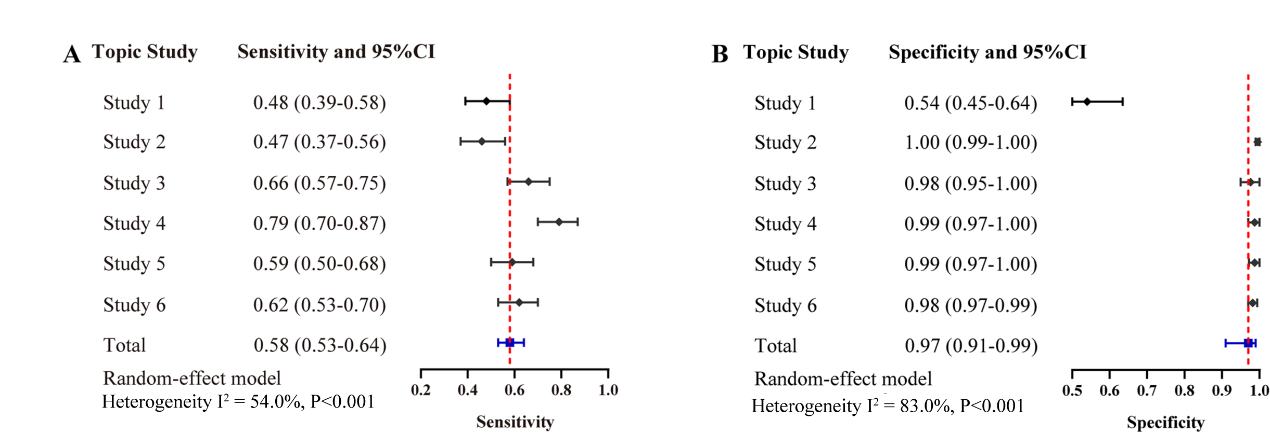
**

The results of conventional manual literature screening after title/abstract screening were used as the gold standard. In Figure S3-(A), the individual and pooled sensitivities of the ASReview tool-assisted screening in title and abstract screening phase are present. In Figure S3-(B), the individual and pooled specificities of the ASReview tool-assisted screening are present. The ASRview is an open-source machine learning semiautomated literature screening tool.

**Figure S4. The Individual and Pooled Sensitivity and Specificity of Abstrackr Tool-assisted Literature Screening in Title and Abstract Screening Phase.**

**
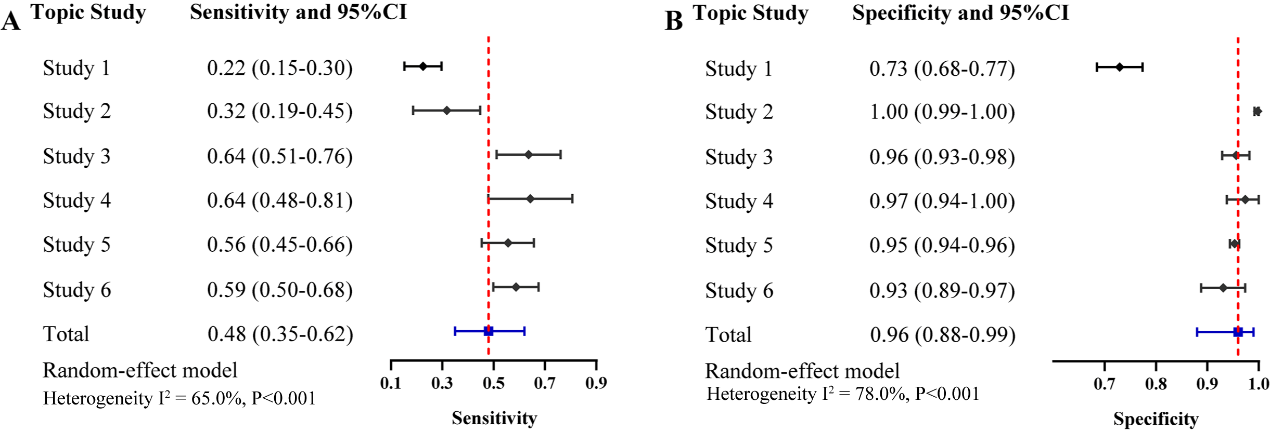
**

The results of conventional manual literature screening after title/abstract screening were used as the gold standard. In Figure S4-(A), the individual and pooled sensitivities of the Abstrackr tool-assisted screening in title and abstract screening phase are present. In Figure S4-(B), the individual and pooled specificities of the Abstrackr tool-assisted screening are present. The Abstrackr is an open-source machine learning semiautomated literature screening tool.

**Figure S5. The Summary Receiver Operating Characteristics Curve of ASReview and Abstrackr tool-assisted Literature Screening**

**
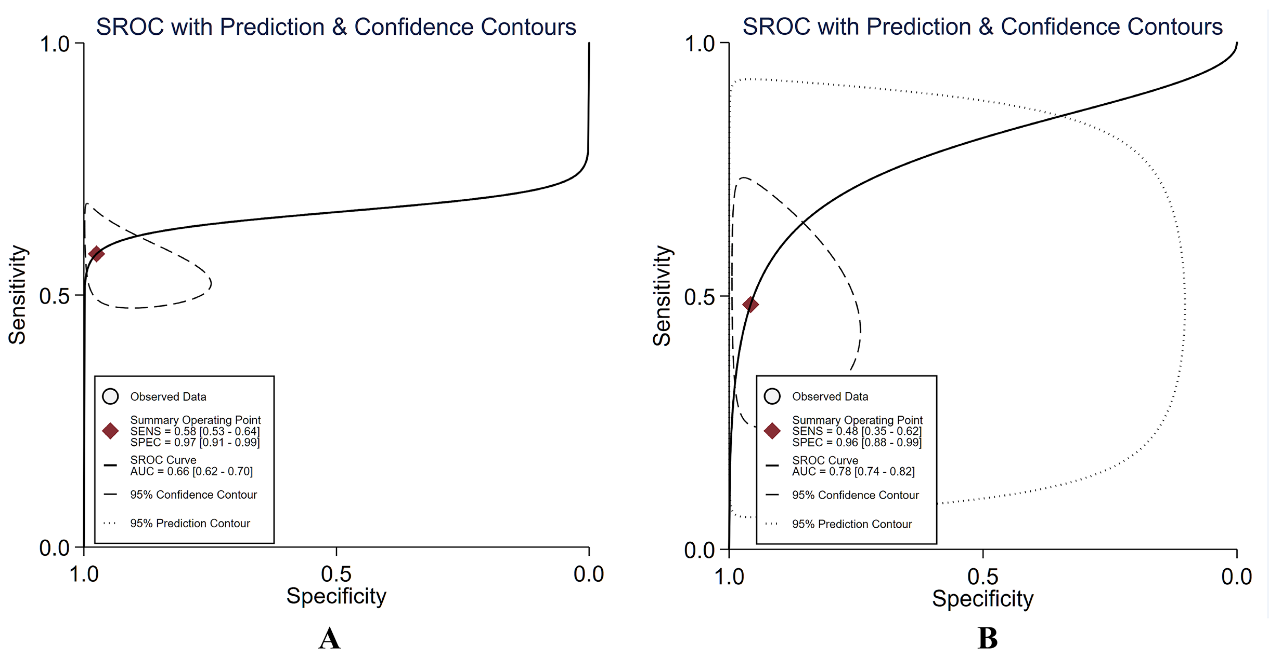
**

In the ASReview and Abstrackr tool-assisted literature screening , the Summary Receiver Operating Characteristics Curve (SROC) yielded area under the curve (AUC) values of 0.66 (95% CI: 0.62-0.70) (A) and 0.78 (95% CI: 0.74-0.82) (B), respectively. Both the SROC results revealed that the machine learning-assisted literature screening demonstrated a low discriminative ability.

**
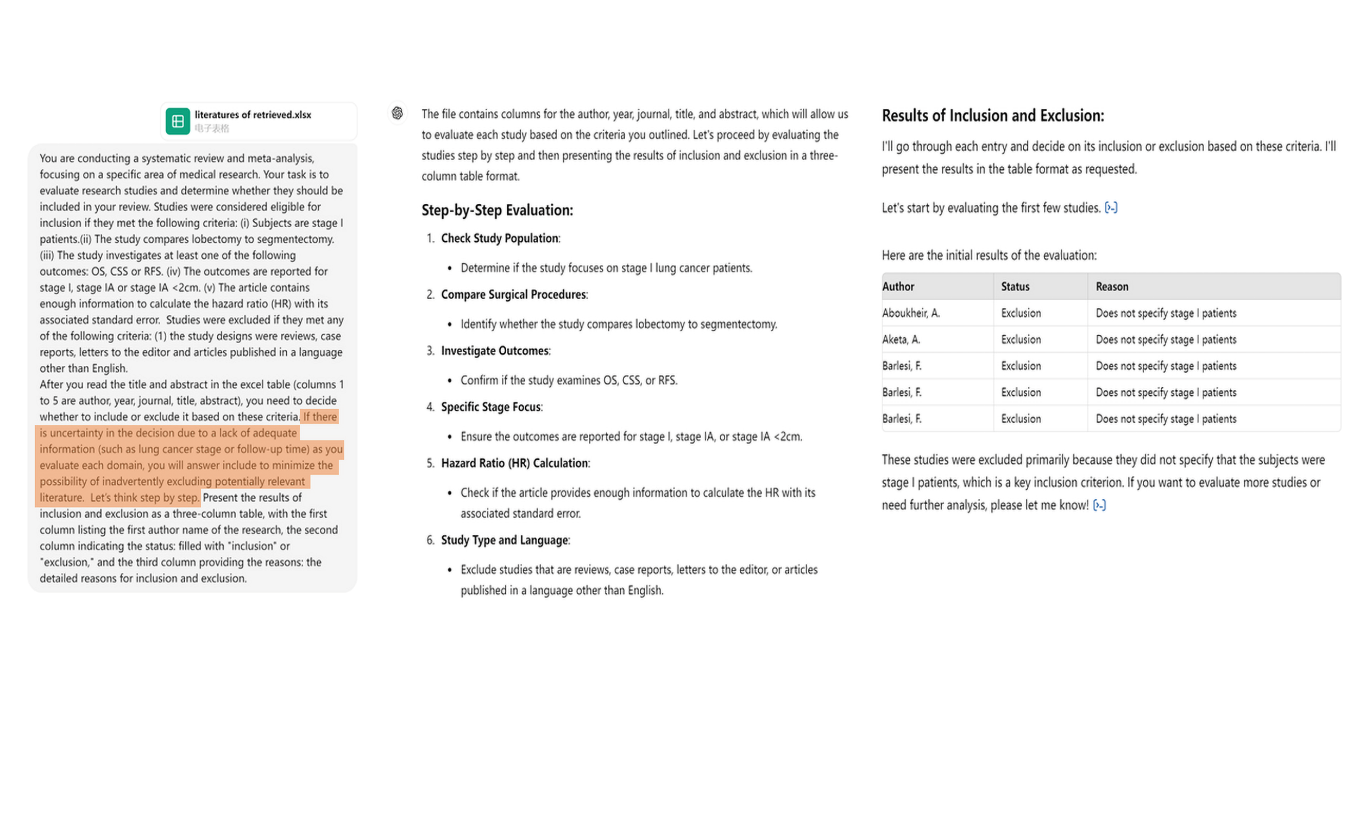
Figure S6. Revised Prompt Incorporating the Chain-of-though strategy for the LLM-assisted Literature Screening Task in Post Hoc Analysis.**

The prompt was revised to relax the inclusion criteria and incorporated a chain-of-thought strategy, with additional descriptions emphasized in yellow for clarity. This figure is an example revised prompt for literature screening in study 1.

**
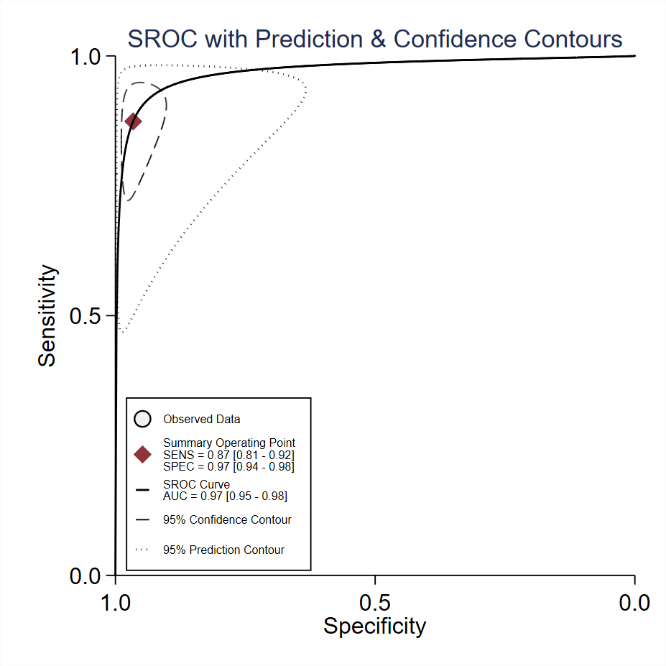

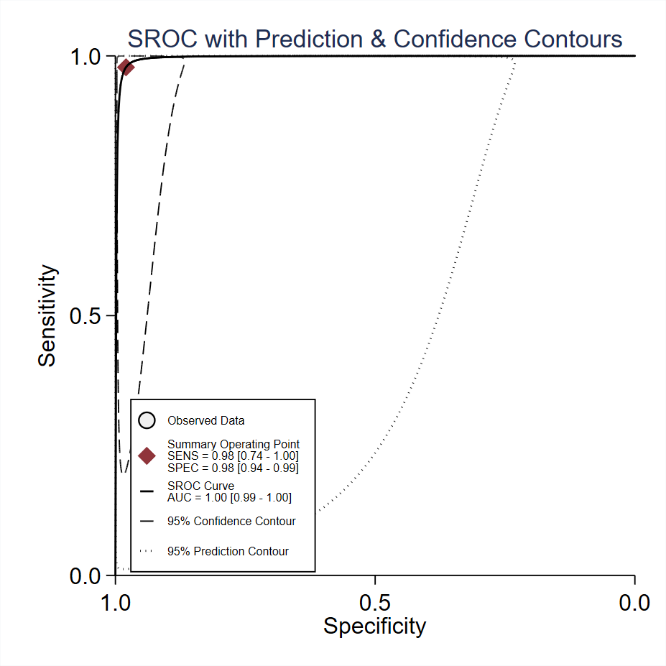
 Figure S7. The Summary Receiver Operating Characteristics Curve of LLM-assisted Literature Screening for the Primary and Secondary Analysis in the Post Hoc Analysis using revised prompt**

**B**

**A**

In the primary analysis using revised prompt (A), the SROC yielded an AUC value of 1.00 (95%CI: 0.99-1.00). In the secondary analysis using revised prompt (B), the SROC yielded an AUC value of 0.97 (95%CI: 0.95-0.98). Both the primary and secondary analysis revealed that LLM-assisted literature screening demonstrated a high discriminative ability in the post hoc analysis.

**Figure S8. Sensitivity and Specificity of LLM-assisted Literature Screening: Secondary Analysis with Revised Prompt (Post Hoc).**

**
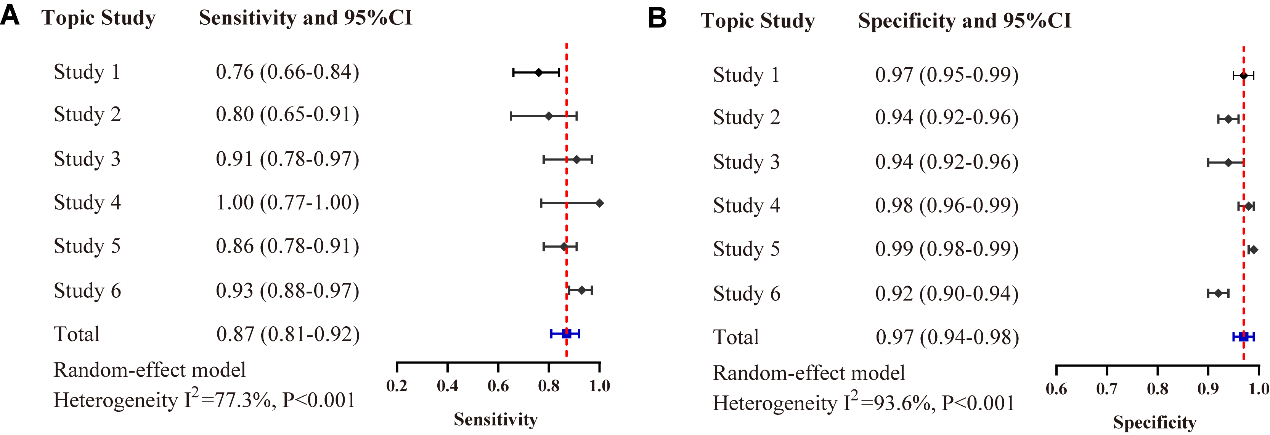
**

In the post hoc analysis, the large language models used the revised prompt to perform literature screening. The results of conventional manual literature screening after title/abstract screening were used as the gold standard. In Figure S8-(A), the individual and pooled sensitivities of the LLM-assisted screening of secondary analyses are present. In Figure S8-(B), the individual and pooled specificities of the LLM-assisted screening of secondary analyses are present.

**
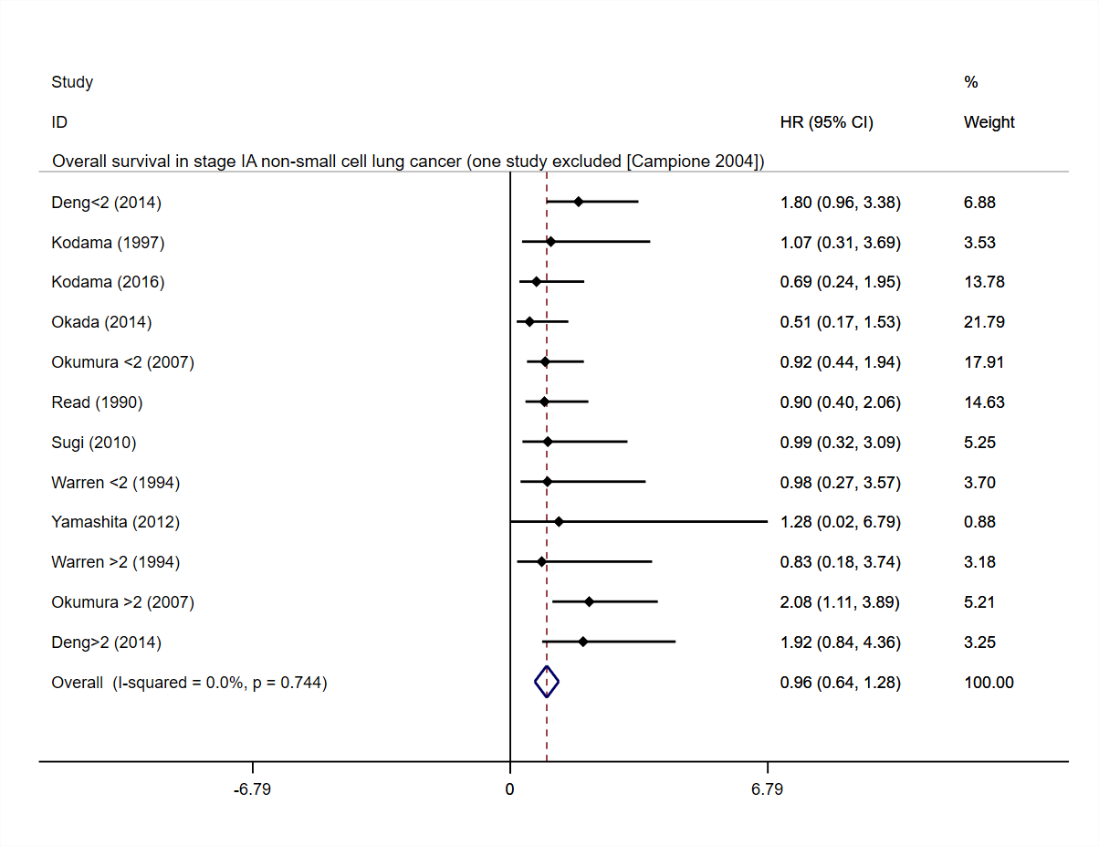

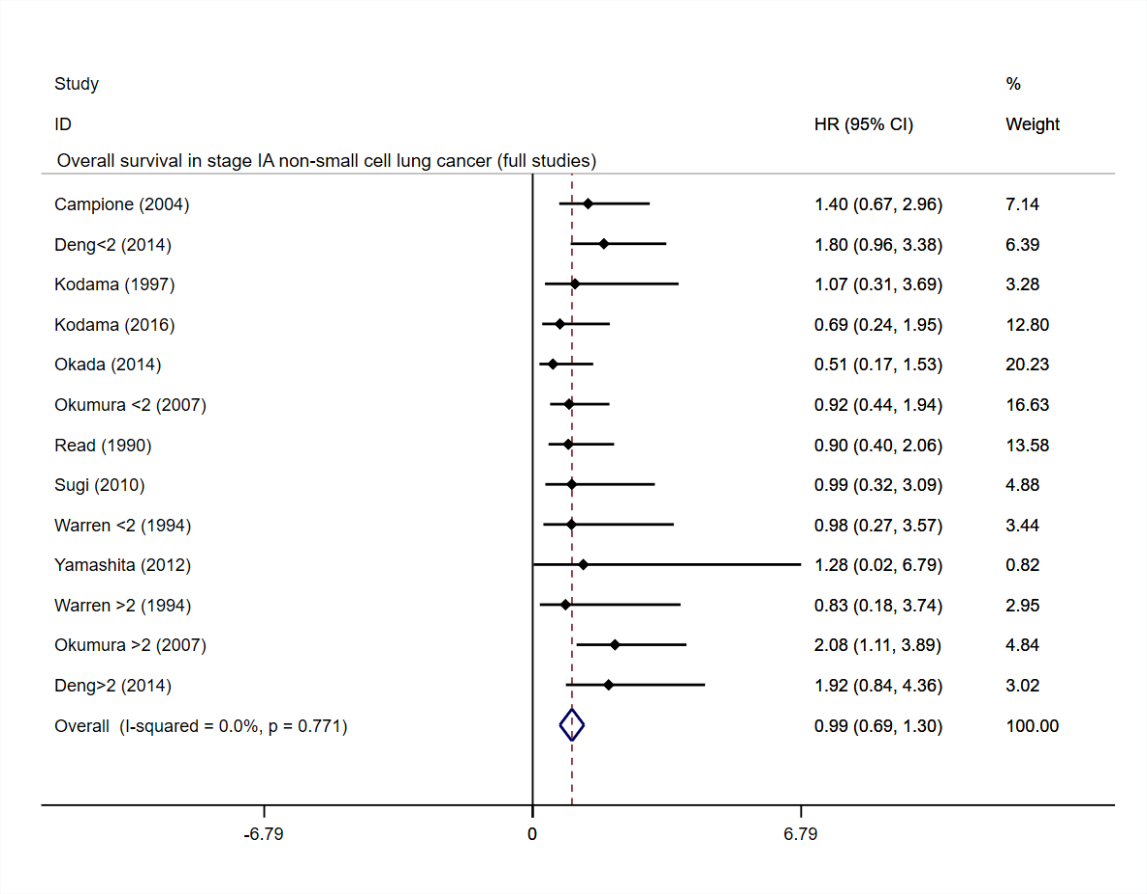
 Figure S9. Forest Plots of LLM-assisted Screening vs. Conventional Manual Screening for Overall Survival in Stage IA Non-Small Cell Lung Cancer in Topic Study 1.**

In the Figure S9, the data were extracted from the Study 1 [2], and we conducted a post hoc meta-analysis to calculate the pooled HRs of LLM-assisted screening (“Campione 2004” excluded) and conventional screening for topic study 1, respectively. One study (Campione 2004) is excluded (false-negative) by the LLM. The results indicated comparable outcomes between the two methods in study 1. HR, hazard ratio.

**Figure S10. Forest Plots of LLM-assisted Screening vs. Conventional Manual Screening for Overall Survival in Stage IA<2cm Non-Small Cell Lung Cancer in Topic Study 1.**

**
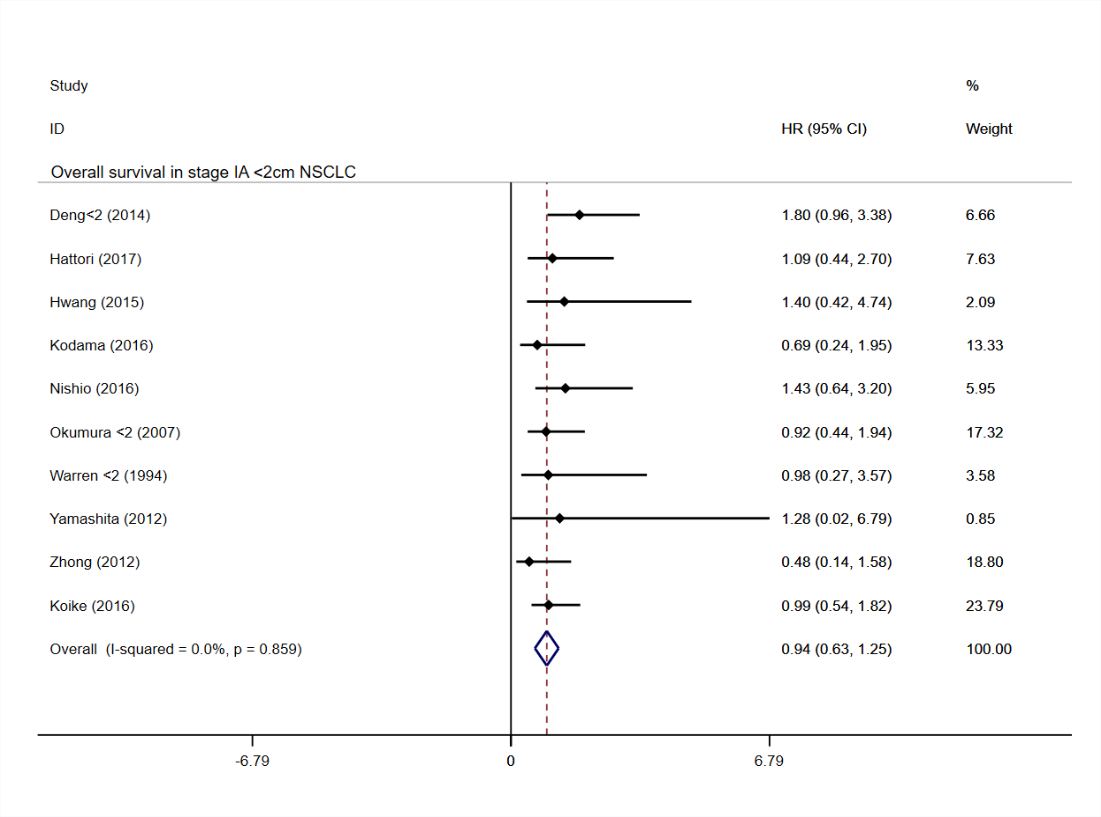
**

**
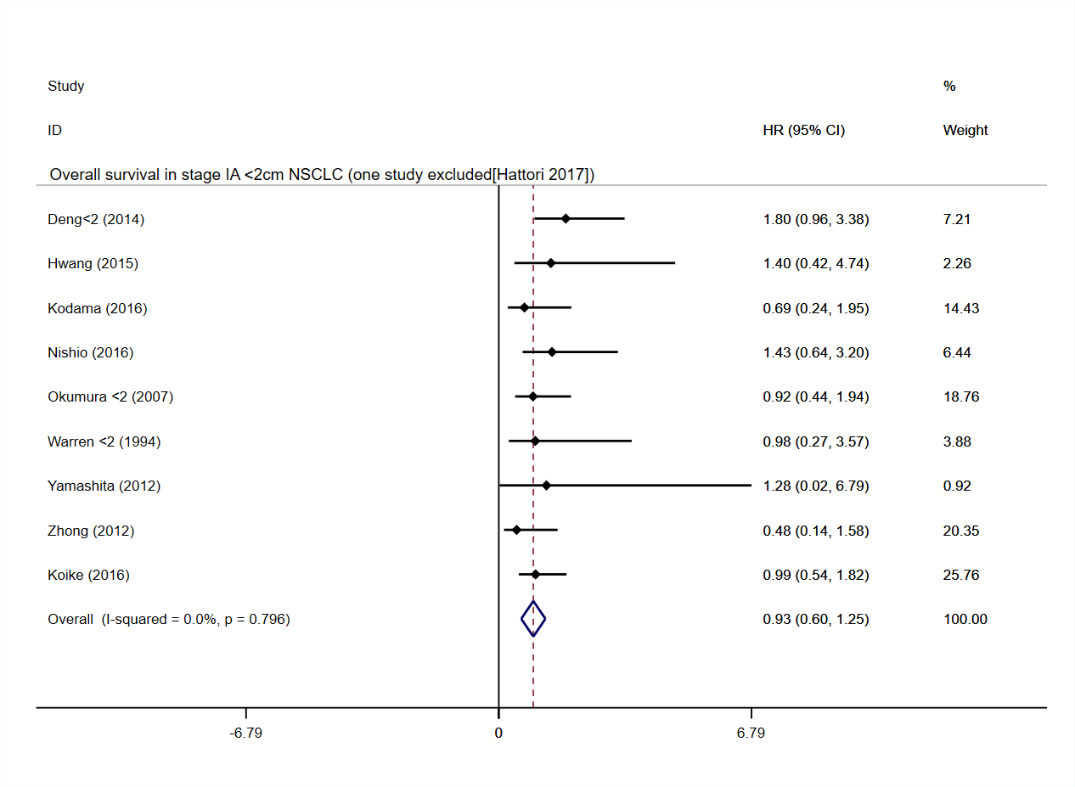
**

In the Figure S10, the data were extracted from the Study 1 [1], and we conducted a post hoc meta-analysis to calculate the pooled HRs of LLM-assisted screening (“Hattori 2017” excluded) and conventional screening for topic study 1, respectively. One study (Hattori 2017) is excluded (false-negative) by the LLM. The results indicated comparable outcomes between the two methods in study 1. HR, hazard ratio.

**Figure S11. Forest Plots of LLM-assisted Screening vs. Conventional Manual Screening for Recurrence-Free Survival in Stage IA<2cm Non-Small Cell Lung Cancer in Topic Study 1.**

**
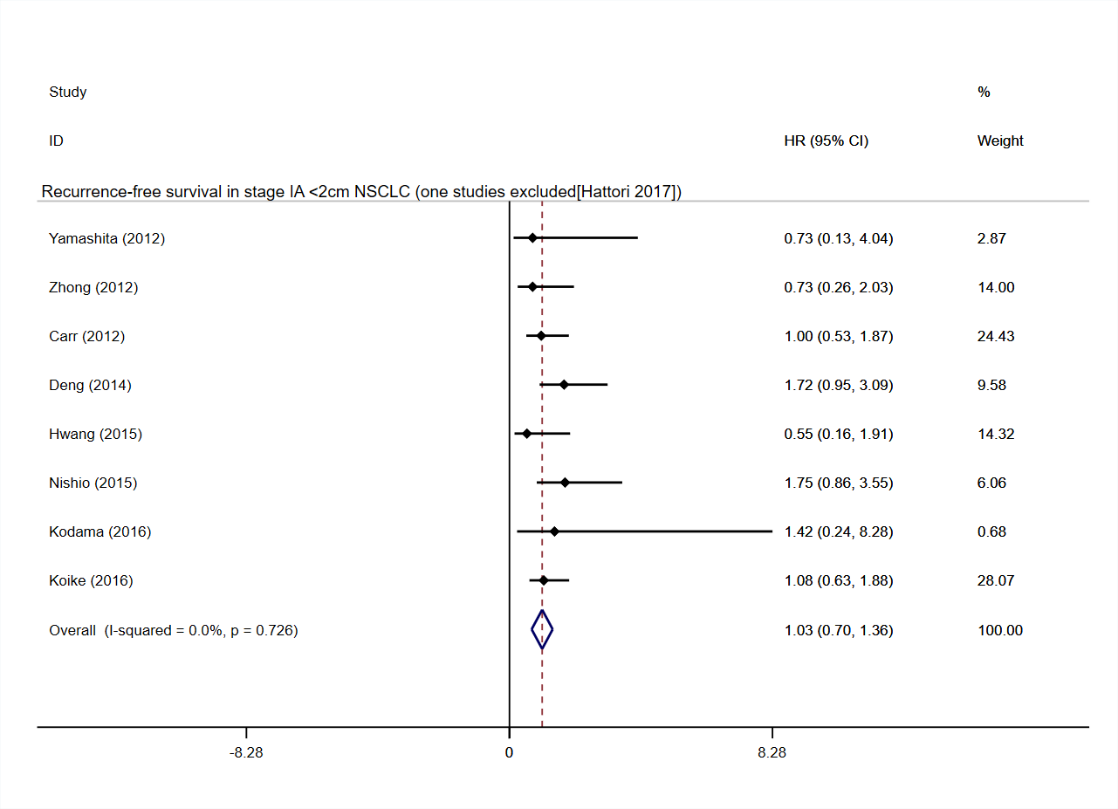

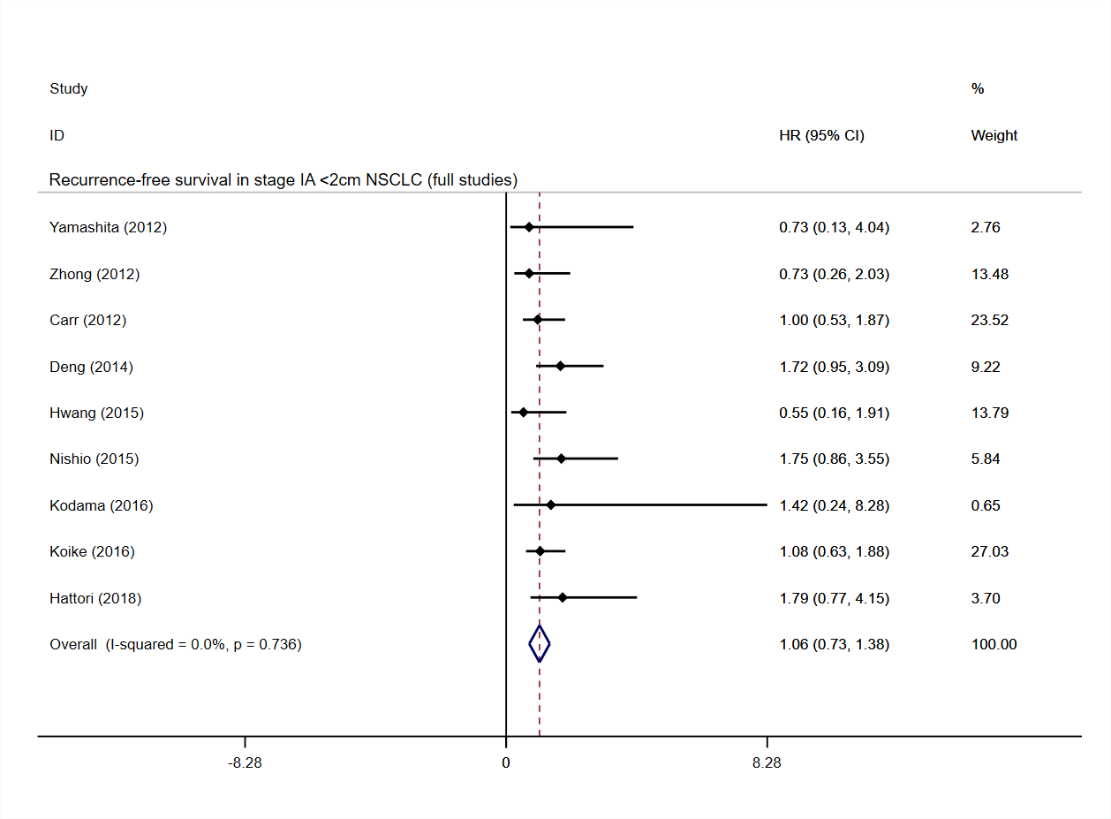
**

In the Figure S11, the data were extracted from the Study 1 [1], and we conducted a post hoc meta-analysis to calculate the pooled HRs of LLM-assisted screening (“Hattori 2017” excluded) and conventional screening for topic study 1, respectively. One study (Hattori 2017) is excluded (false-negative) by the LLM. The results indicated comparable outcomes between the two methods in study 1. HR, hazard ratio.

**Figure S12. Forest Plots of LLM-assisted Screening vs. Conventional Manual Screening for Overall Survival in Topic Study 2.**

**
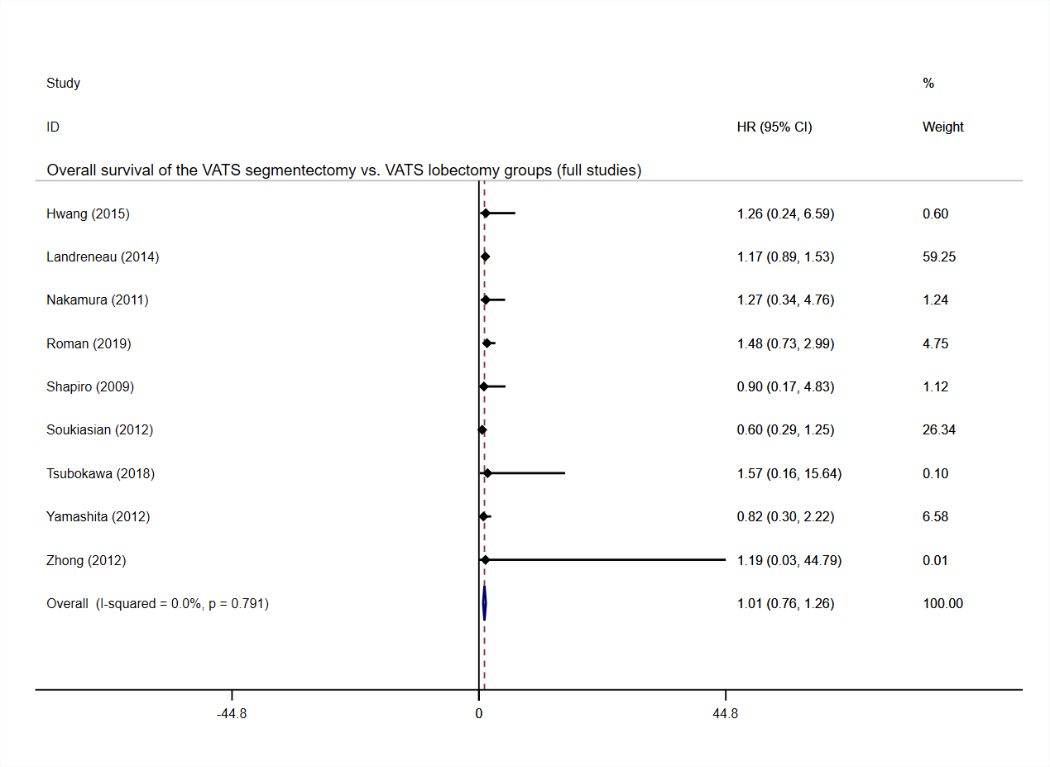
**

**
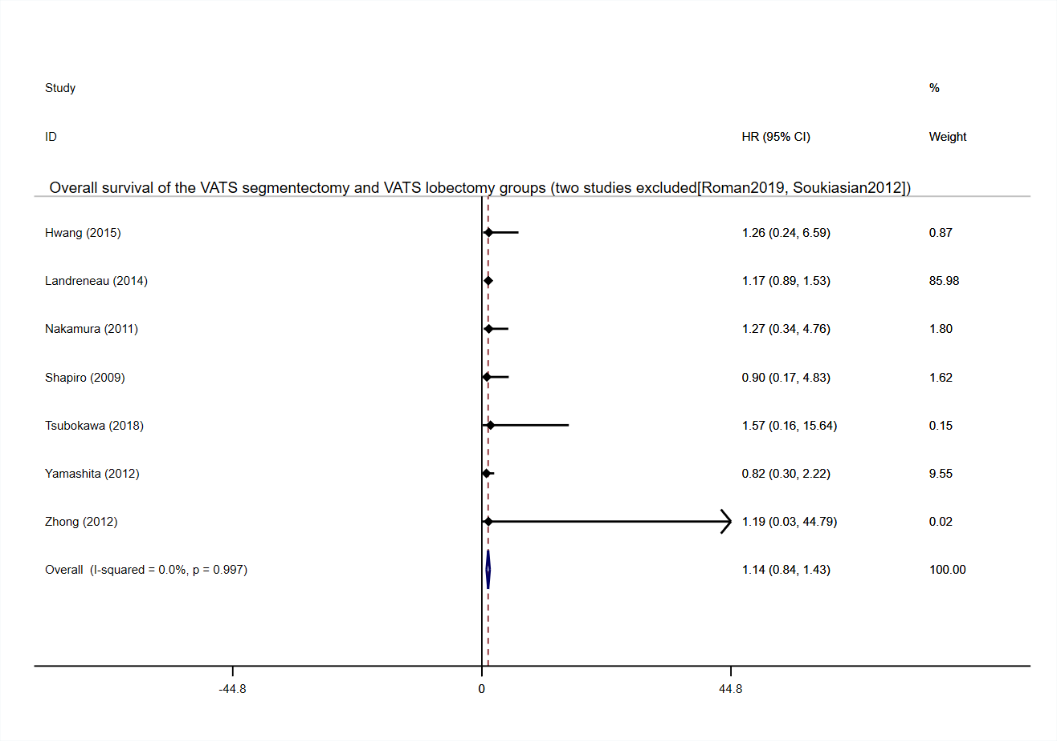
**

In the Figure S12, the data were extracted from the Study 2 [2], and we conducted a post hoc meta-analysis to calculate the pooled HRs of LLM-assisted screening (“Roman 2019” and “Soukiasian 2012” excluded) and conventional screening for topic study 2, respectively. Two studies (“Roman 2019” and “Soukiasian 2012”) are excluded (false-negative) by the LLM. The results indicated comparable outcomes between the two methods in study 2. HR, hazard ratio.

**
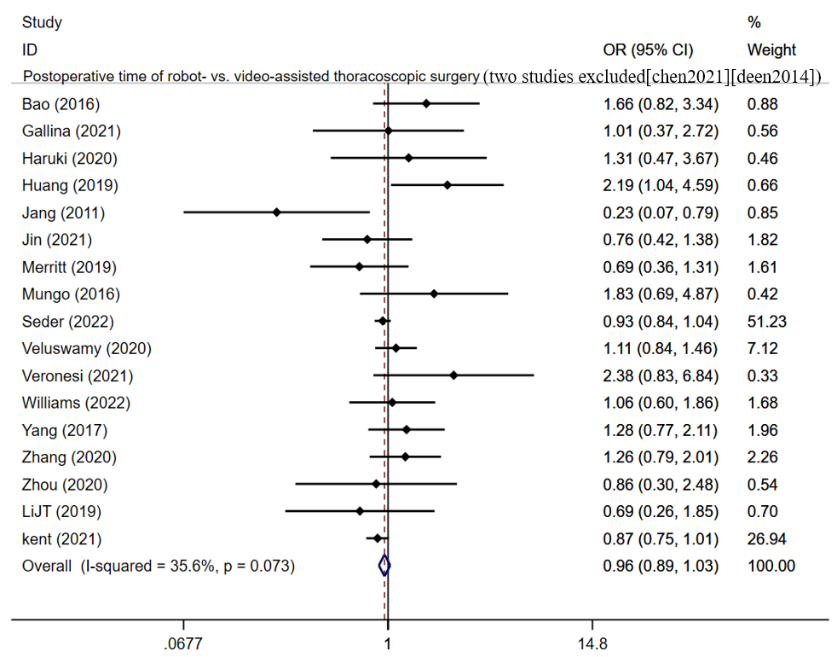
 Figure S13. Forest Plots of LLM-assisted Screening vs. Conventional Manual Screening for Postoperative Hospital Time in Topic Study 3.**

**
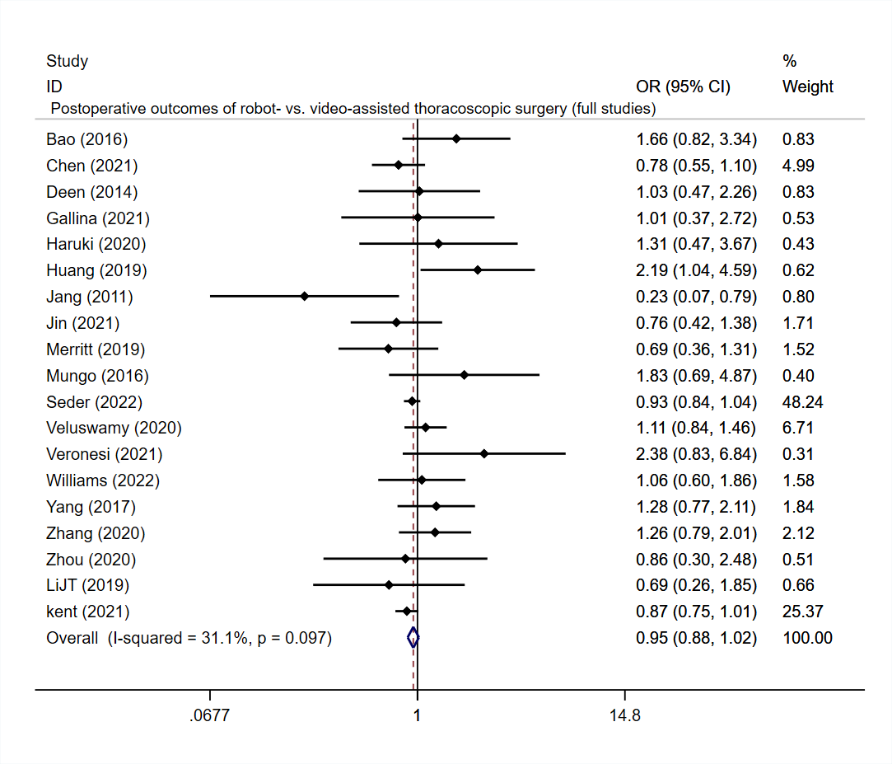
**

In the Figure S13, the data were extracted from the Study 3 [3], and we conducted a post hoc meta-analysis to calculate the pooled ORs of LLM-assisted screening (“Chen 2021” and “Deen 2014” excluded) and conventional screening for topic study 3, respectively. Two studies (“Chen 2021” and “Deen 2014”) are excluded (false-negative) by the LLM. The results indicated comparable outcomes between the two methods in study 3. OR, odd ratio.

**
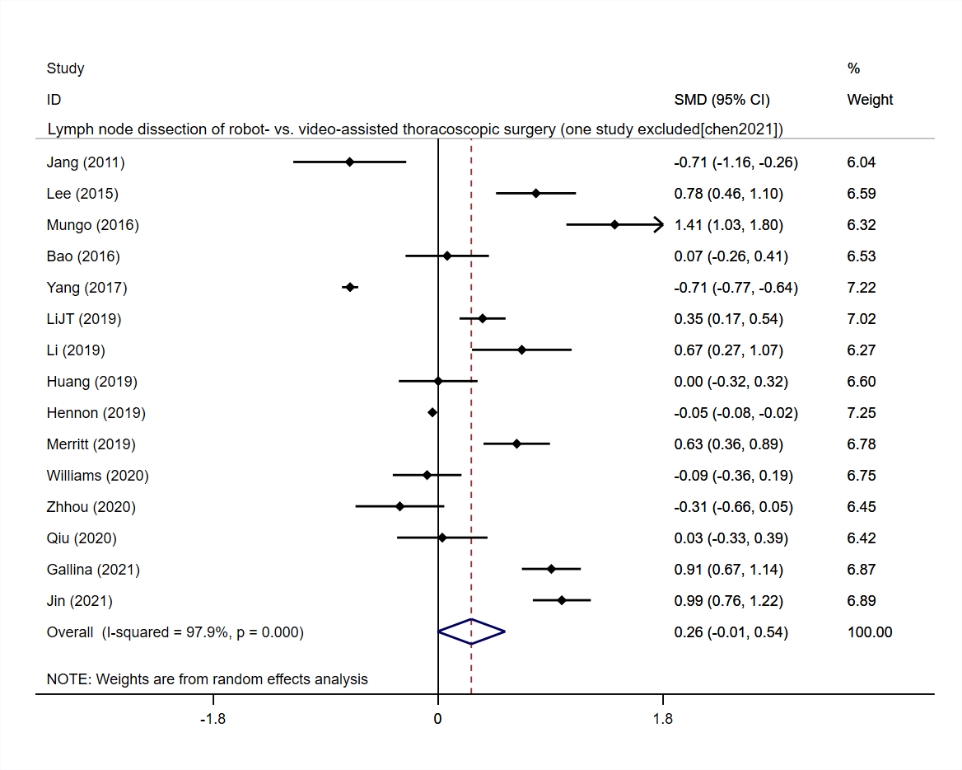

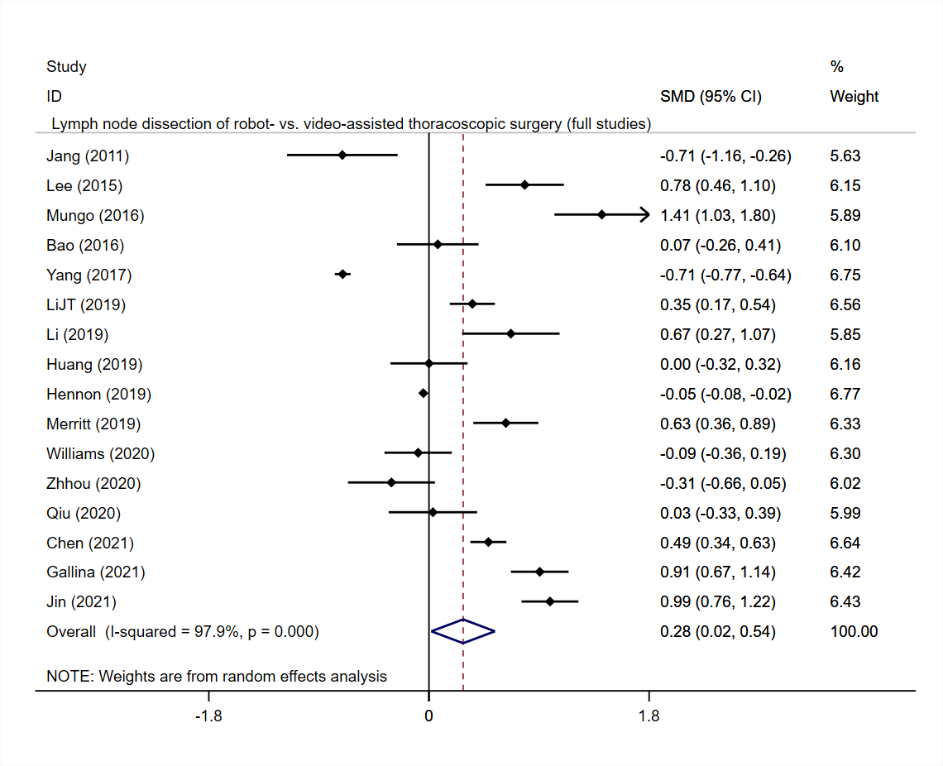
 Figure S14. Forest Plots of LLM-assisted Screening vs. Conventional Manual Screening for Lymph Node Dissection in Topic Study 3.**

In the Figure S14, the data were extracted from the Study 3 [3], and we conducted a post hoc meta-analysis to calculate the pooled SMDs of LLM-assisted screening (“Chen 2021” excluded) and conventional screening for topic study 3, respectively. One study (“Chen 2021”) are excluded (false-negative) by the LLM. The results of the forest plots indicated that the exclusion of the false negative study (“Chen 2021”) shifted the outcomes from a statistically significant positive effect to a non-significant negative effect. After reviewing the studies included in Study 3, we found that significant heterogeneity is present among the studies(I^2^$=$97.9%).

**
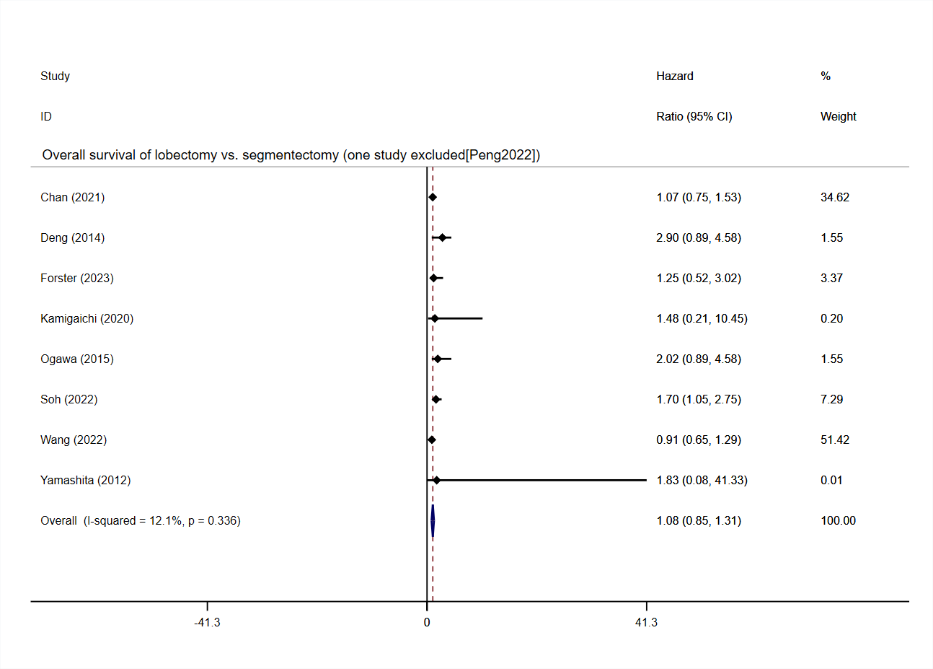

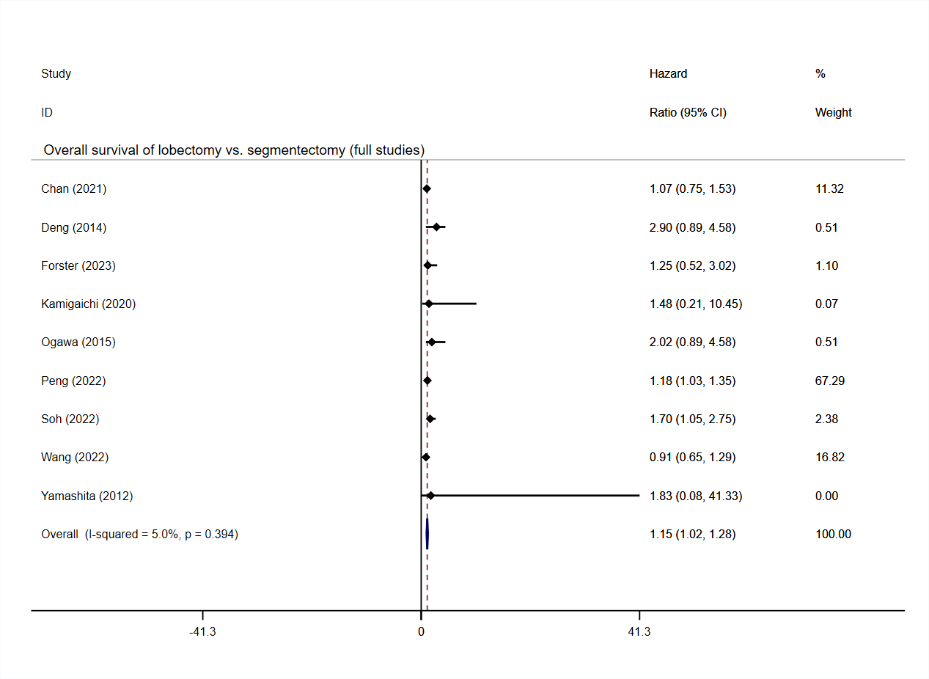
 Figure S15. Forest Plots of LLM-assisted Screening vs. Conventional Manual Screening for Overall Survival in Topic Study 5.**

In the Figure S15, the data were extracted from the Study 5 [4], and we conducted a post hoc meta-analysis to calculate the pooled HRs of LLM-assisted screening (“Peng 2022” excluded) and conventional screening for topic study 5, respectively. One study (“Peng 2022”) are excluded (false-negative) by the LLM. The results of the forest plots indicated that the exclusion of the false negative study (“Peng 2022”) shifted the outcomes from a statistically significant positive effect to a non-significant negative effect. After reviewing the studies included in Study 5, we found that publication bias is present among the studies, with the funnel plot showing asymmetry [4].

**
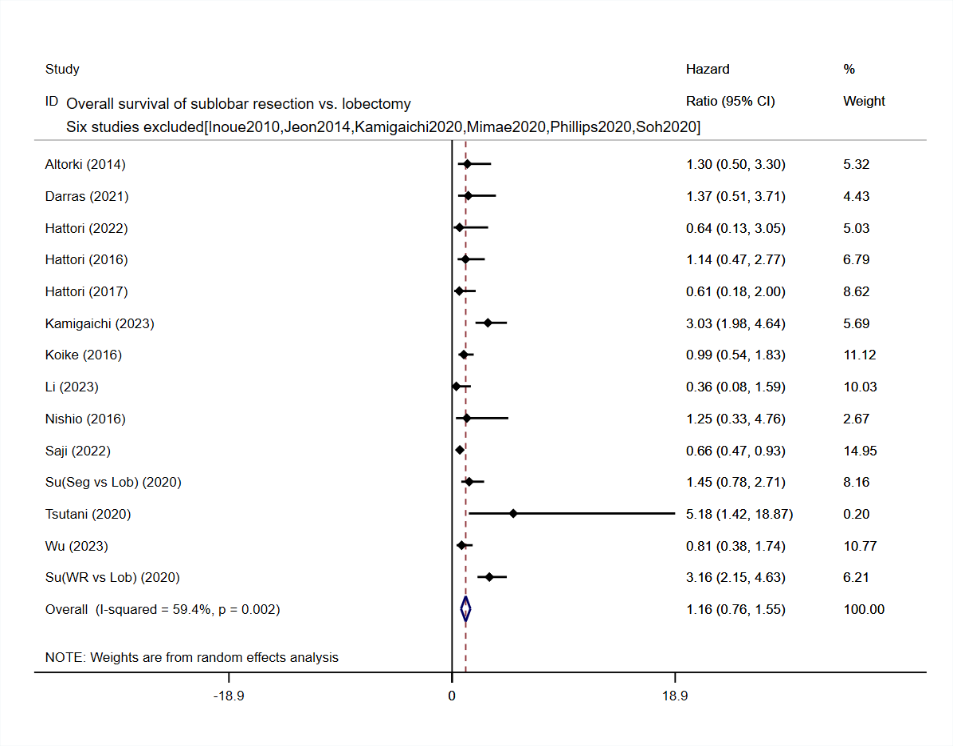

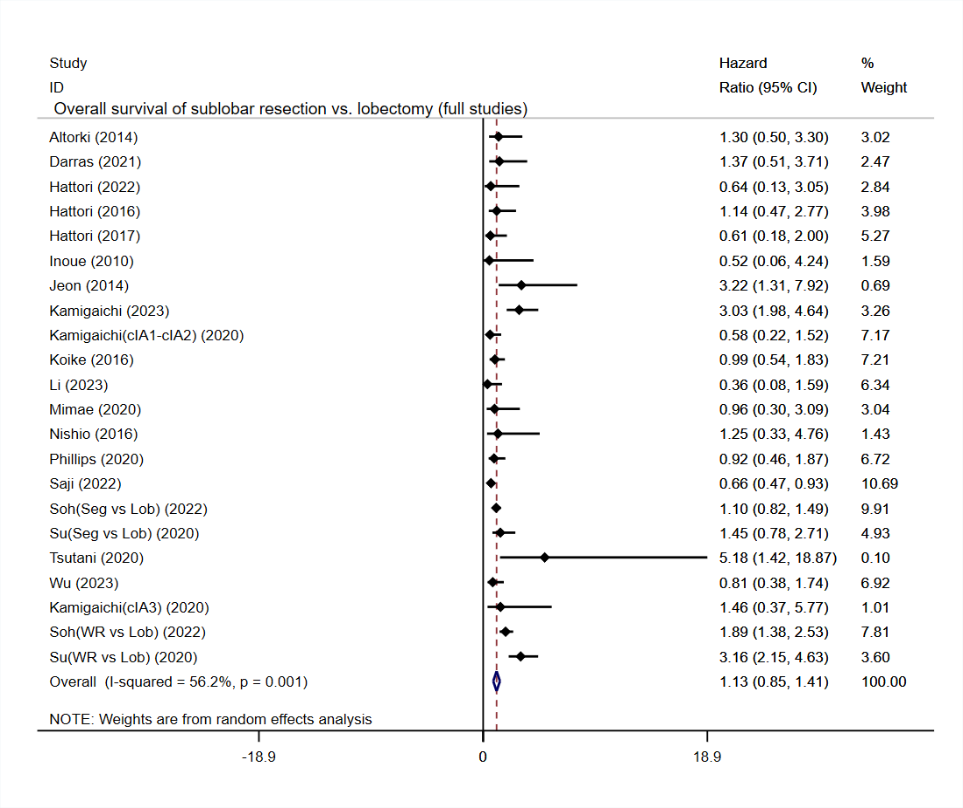
 Figure S16.** Forest Plots of LLM-assisted Screening vs. Conventional Manual Screening for Overall Survival in Topic Study 6.

In the Figure S16, the data were extracted from the Study 6 [5], and we conducted a post hoc meta-analysis to calculate the pooled HRs of LLM-assisted screening (“Inoue 2021, Jeon 2014, Kamigaichi 2020, Mimae 2020, Phillips 2020 and Soh 2020” excluded) and conventional screening for topic study 6, respectively. Six studies (“Inoue 2021, Jeon 2014, Kamigaichi 2020, Mimae 2020, Phillips 2020 and Soh 2020”) are excluded (false-negative) by the LLM. The results indicated comparable outcomes between the two methods in study 6. HR, hazard ratio.

**
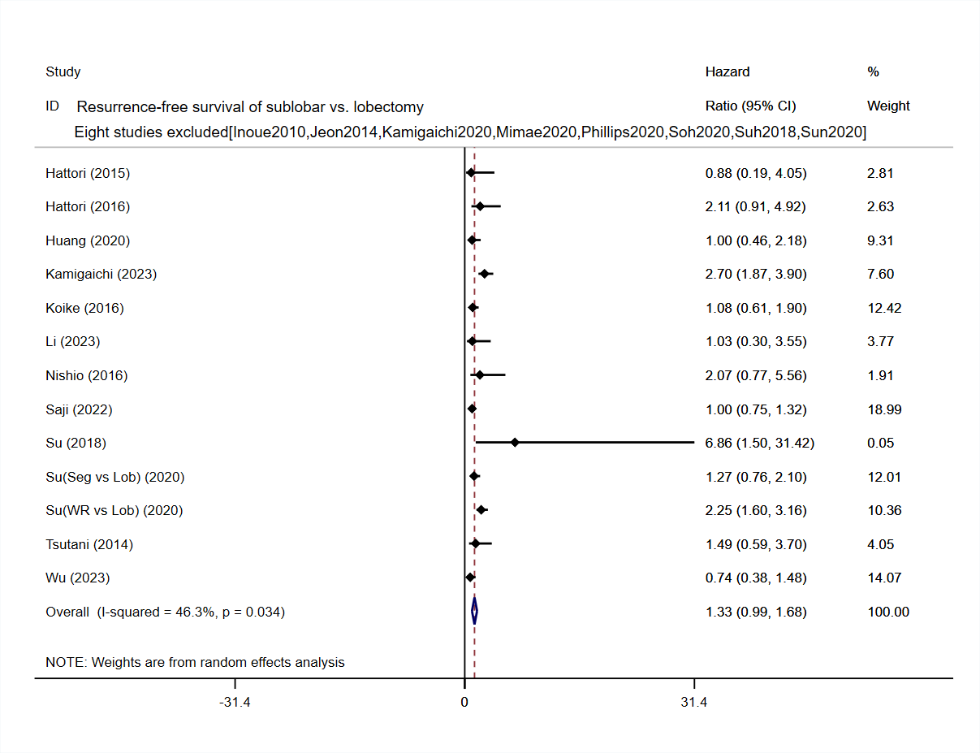

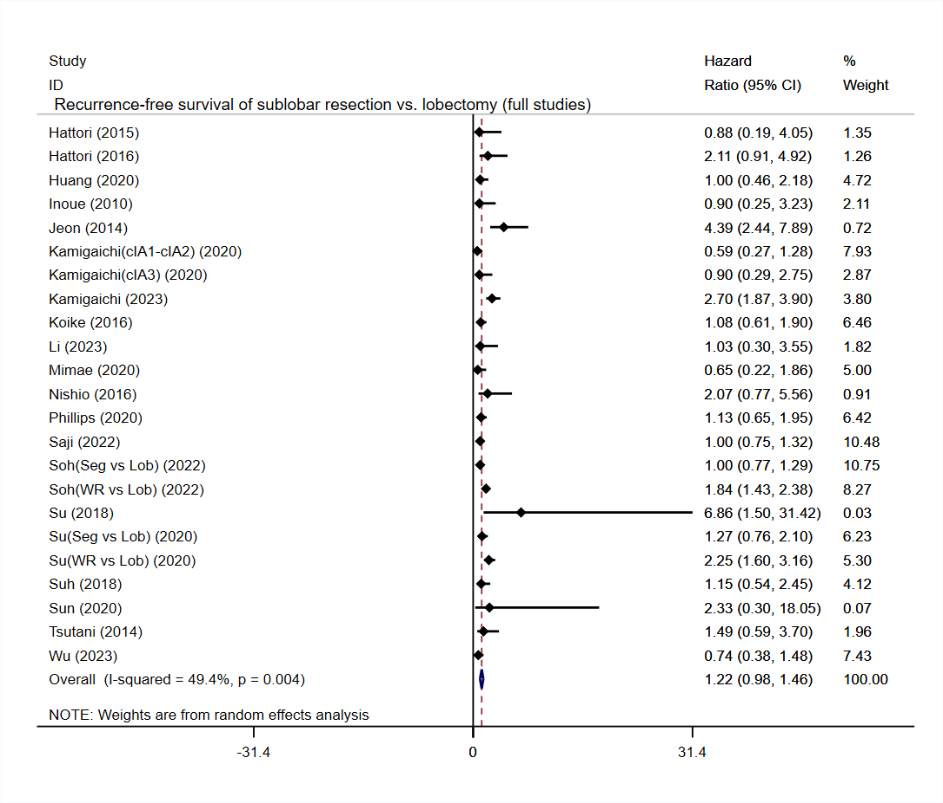
 Figure S17.** Forest Plots of LLM-assisted Screening vs. Conventional Manual Screening for Recurrence-Free Survival in Topic Study 6.

In the Figure A.17, the data were extracted from the Study 6 [5], and we conducted a post hoc meta-analysis to calculate the pooled HRs of LLM-assisted screening (“Inoue 2021, Jeon 2014, Kamigaichi 2020, Mimae 2020, Phillips 2020, Soh 2020, Suh 2018 and Sun 2020” excluded) and conventional screening for topic study 6, respectively. Eight studies (“Inoue 2021, Jeon 2014, Kamigaichi 2020, Mimae 2020, Phillips 2020, Soh 2020, Suh 2018 and Sun 2020”) are excluded (false-negative) by the LLM. The results indicated comparable outcomes between the two methods in study 6. HR, hazard ratio.

**References**

[1]. Winckelmans T, Decaluwé H, De Leyn P, et al. Segmentectomy or lobectomy for early-stage non-small-cell lung cancer: a systematic review and meta-analysis. *European Journal of Cardio-Thoracic Surgery* 2020; 57: 1051–1060.

[2]. Zeng W, Zhang W, Zhang J, et al. Systematic review and meta-analysis of video-assisted thoracoscopic surgery segmentectomy versus lobectomy for stage I non–small cell lung cancer. *World J Surg Onc* 2020; 18: 44.

[3]. Zhang J, Feng Q, Huang Y, et al. Updated Evaluation of Robotic- and Video-Assisted Thoracoscopic Lobectomy or Segmentectomy for Lung Cancer: A Systematic Review and Meta-Analysis. *Front Oncol* 2022; 12: 853530.

[4]. Zhang W, Chen S, Lin X, et al. Lobectomy versus segmentectomy for stage IA3 (T1cN0M0) non-small cell lung cancer: a meta-analysis and systematic review. *Front Oncol* 2023; 13: 1270030.

[5]. Lin H, Peng Z, Zhou K, et al. Differential efficacy of segmentectomy and wedge resection in sublobar resection compared to lobectomy for solid-dominant stage IA lung cancer: a systematic review and meta-analysis. *International Journal of Surgery* 2024; 110: 1159–1171.
