# Supplementary material for: Accuracy of Large Language Models for Literature Screening in Thoracic Surgery: Diagnostic Study
Source: J Med Internet Res. 2025 Mar 11;27:e67488. doi: 10.2196/67488 (PMC11937709; doi:10.2196/67488)
Supplement: Multimedia Appendix 2 [file jmir_v27i1e67488_app2.docx]

**Appendix 2**

**Table of Contents:**

1. **All detailed content of LLM prompts (Take study 1 screening as an example)**
2. **The detailed steps and procedures of machine learning-assisted literature screening.**
3. **The results of LLM-assisted literature screening process.**
4. **The sensitivity and specificity of LLM-assisted screening in secondary analysis using original prompt.**
5. **Revised prompt**
6. **The sensitivity and specificity of LLM-assisted screening in primary analysis using revised prompt in post hoc analysis.**
7. **The sensitivity, specificity and area under the curve of the LLM-assisted screening of secondary analysis using revised prompt in post hoc analysis.**
8. **Post hoc meta-analysis of index results of LLM-assisted screening vs. conventional manual screening.**

**Supplemental Material Content**

**S1.All detailed content of LLM prompts (Take study 1 screening as an example)**

You are conducting a systematic review and meta-analysis, focusing on a specific area of medical research. Your task is to evaluate research studies and determine whether they should be included in your review.

Studies were considered eligible for inclusion if they met the following criteria:

(i) Subjects are stage I patients.

(ii) The study compares lobectomy to segmentectomy.

(iii) The study investigates at least one of the following outcomes: OS, CSS or RFS.

(iv) The outcomes are reported for stage I, stage IA or stage IA <2cm.

(v) The article contains enough information to calculate the hazard ratio (HR) with its associated standard error.

Studies were excluded if they met any of the following criteria:

(i) The study designs were reviews, case reports, letters to the editor

(ii) Articles published in a language other than English.

**(1). At initial title and abstract screening phase**

After you read the title and abstract in the excel table (columns 1 to 5 are author, year, journal, title, abstract), you need to decide whether to include or exclude it based on these criteria. Present the results of inclusion and exclusion as a three-column table, with the first column listing the first-author name of the research, the second column indicating the status: filled with "inclusion" or "exclusion," and the third column providing the reasons: the detailed reasons for inclusion and exclusion.

**(2). At initial full-text screening phase**

After you read the Full-text word document, you need to decide whether to include or exclude it based on these criteria. Present the results of inclusion and exclusion, and providing the specific reasons for inclusion and exclusion.

**(3). At post hoc title and abstract screening phase**

After you read the title and abstract in the excel table (columns 1 to 5 are author, year, journal, title, abstract), you need to decide whether to include or exclude it based on these criteria. If there is uncertainty in the decision due to a lack of adequate information (such as lung cancer stage or follow-up time) as you evaluate each domain, you will answer include to minimize the possibility of inadvertently excluding potentially relevant literature. Let’s think step by step. Present the results of inclusion and exclusion as a three-column table, with the first column listing the first-author name of the research, the second column indicating the status: filled with "inclusion" or "exclusion," and the third column providing the reasons: the detailed reasons for inclusion and exclusion.

**(4). At post hoc full-text screening phase**

After you read the Full-text word document, you need to decide whether to include or exclude it based on these criteria. If there is uncertainty in the decision due to a lack of adequate information as you evaluate each domain, you will answer include to minimize the possibility of inadvertently excluding potentially relevant literature. Let’s think step by step. Present the results of inclusion and exclusion, and providing the specific reasons for inclusion and exclusion.

**Note:** The underlined sections represent the inclusion and exclusion criteria for Study 1. The instructions for Studies 2 to 6 were similar to those for Study 1, except that the inclusion and exclusion criteria had to be modified.

**S2. The detailed steps and procedures of machine learning-assisted literature screening.**

We selected two representative machine learning screening tools: ASReview^1^ ([https://asreview.nl/download/#](https://asreview.nl/download/)) and Abstrackr^2^ (<http://abstrackr.cebm.brown.edu/>). The reasons for choosing these tools are primarily: 1. They are open-source, free, and have been validated and evaluated with external data^1-4^. 2. Both have user-friendly interfaces, low learning curves, and are easily scalable^1,2^. 3. They allow users to download the predicted relevant studies for further review.

Procedure and Steps: First, the records retrieved from Study 1, containing titles and abstracts, are uploaded to the user interface in RIS or xlsx format. Reviewers manually assess whether each study is "relevant" or "irrelevant" to the research topic and label them accordingly. This process is repeated iteratively. Based on the labeled data, the active learning model then begins predicting and labeling the remaining studies, classifying them as "relevant," "irrelevant," or "unsure." In ASReview, the reviewer labeled a total of 20 studies (1 relevant and 19 irrelevant) to train the model^3^. In Abstrackr, the reviewer labeled 40 studies before activating the active learning model to predict and label relevant studies^4^. Ultimately, we included studies marked as "relevant" and "unsure" as entries for full-text screening^4^. The same process was repeated for Studies 2-6.

The screening results are presented in Table S4 in Appendix 1. The individual sensitivity and specificity of ASReview-assisted screening for Studies 1 through 6 as follows: 0.48 (95% CI: 0.39-0.58) and 0.54 (95% CI: 0.45-0.64), 0.47 (95% CI: 0.37-0.56) and 1.00 (95% CI: 0.99-1.00), 0.66 (95% CI: 0.57-0.75) and 0.98 (95% CI: 0.95-1.00), 0.79 (95% CI: 0.70-0.87) and 0.99 (95% CI: 0.97-1.00), 0.59 (95% CI: 0.50-0.68) and 0.99 (95% CI: 0.97-1.00), 0.62 (95% CI: 0.53-0.70) and 0.98 (95% CI: 0.97-0.99), respectively (Figure S3 in Appendix 1). The individual sensitivity and specificity of Abstrackr-assisted screening for Studies 1 through 6 as follows: 0.22 (95% CI: 0.15-0.30) and 0.73 (95% CI: 0.68-0.77), 0.32 (95% CI: 0.19-0.45) and 1.00 (95% CI: 0.99-1.00), 0.64 (95% CI: 0.51-0.76) and 0.96 (95% CI: 0.93-0.98), 0.64 (95% CI: 0.48-0.81) and 0.97 (95% CI: 0.94-1.00), 0.56 (95% CI: 0.45-0.66) and 0.95 (95% CI: 0.94-0.96), 0.59 (95% CI: 0.50-0.68) and 0.93 (95% CI: 0.89-0.97), respectively (Figure S4 in Appendix 1).

**S3.The results of LLM-assisted literature screening process.**

Following deduplication in the LLM-assisted literature screening process, the initial search yielded 357, 462, 296, 429, 2,298, and 696 articles for Studies 1 through 6, respectively. Title and abstract screening resulted in 37, 24, 37, 13, 94, and 108 articles selected for full-text review in the corresponding studies. Ultimately, 26, 9, 24, 11, 9, and 18 articles from studies 1 through 6, respectively, met the inclusion criteria and were incorporated into the final meta-analysis.

**S4.The sensitivity and specificity of LLM-assisted screening in secondary analysis using original prompt.**

In the LLM-assisted literature screening process, a total of 37, 24, 37, 13, 94, and 108 articles from Studies 1 through 6, respectively, were included in the secondary analysis (Table S4 in Appendix 1). The secondary analysis revealed the sensitivity and specificity of the LLM-assisted screening for Studies 1 through 6 as follows: 0.38 (95% CI: 0.28-0.48) and 0.93 (95% CI: 0.89-0.96), 0.59 (95% CI: 0.42-0.74) and 1.00 (95% CI: 0.99-1.00), 0.84 (95% CI: 0.70-0.93) and 0.98 (95% CI: 0.96-1.00), 0.93 (95% CI: 0.66-1.00) and 1.00 (95% CI: 0.98-1.00), 0.75 (95% CI: 0.66-0.82) and 0.98 (95% CI: 0.98-0.99), 0.78 (95% CI: 0.70-0.85) and 0.98 (95% CI: 0.96-0.99), respectively (Figure 3). The pooled sensitivity and specificity across these six studies were 0.73 (95% CI: 0.57-0.85) and 0.99 (95% CI: 0.97-0.99), respectively.

**S5.Revised prompt**

During the post-hoc analysis, false-negative results from studies 1, 2, 3, 5, and 6 were reviewed (Table S2 and S3 in Appendix 1). We found that a positive correlation between the complexity of inclusion criteria and the likelihood of LLMs making "exclude" decisions. LLMs exhibited a tendency to strictly adhere to the inclusion criteria specified in the prompts. Conversely, human reviewers typically apply inclusion criteria more conservatively during the initial title and abstract screening phase to minimize the risk of overlooking potentially relevant studies. Based on this observation, it was determined that minor discrepancies between the inclusion criteria in the prompts and the titles or abstracts could be tolerated. Consequently, the prompts were revised to relax the inclusion criteria and incorporated a chain-of-thought strategy (Figure S6 in Appendix 1 and Supplemental Material Content 1 in Appendix 2).

**S6.The sensitivity and specificity of LLM-assisted screening using revised prompt in the primary analysis of post hoc analysis.**

In the post hoc analysis, the primary analysis revealed the sensitivity and specificity of the LLM-assisted screening using revised prompt for Studies 1 through 6 as follows: 1.00 (95% CI: 0.88-1.00) and 0.85 (95% CI: 0.80-0.89), 0.83 (95% CI: 0.52-0.98) and 1.00 (95% CI: 0.98-1.00), 1.00 (95% CI: 0.87-1.00) and 0.97 (95% CI: 0.94-0.99), 1.00 (95% CI: 0.72-1.00) and 0.99 (95% CI: 0.98-1.00), 1.00 (95% CI: 0.69-1.00) and 0.99 (95% CI: 0.99-1.00), 0.85 (95% CI: 0.65-0.96) and 0.96 (95% CI: 0.93-0.97), respectively (Figure 4). The pooled sensitivity and specificity across these six studies were 0.98 (95% CI: 0.74-1.00) and 0.98 (95% CI: 0.94-0.99), respectively.

**S7.The sensitivity, specificity and area under the curve of the LLM-assisted screening using revised prompt in the secondary analysis of post hoc analysis.**

In the post hoc analysis, the secondary analysis revealed the sensitivity and specificity of the LLM-assisted screening using revised prompt for Studies 1 through 6 as follows: 0.76 (95% CI: 0.66-0.84) and 0.97 (95% CI: 0.95-0.99), 0.80 (95% CI: 0.65-0.91) and 0.94 (95% CI: 0.92-0.96), 0.91 (95% CI: 0.78-0.97) and 0.94 (95% CI: 0.92-0.96), 1.00 (95% CI: 0.77-1.00) and 0.98 (95% CI: 0.96-0.99), 0.86 (95% CI: 0.78-0.91) and 0.99 (95% CI: 0.98-0.99), 0.93 (95% CI: 0.88-0.97) and 0.92 (95% CI: 0.90-0.94), respectively (Figure S8 in Appendix 1). The pooled sensitivity and specificity across these six studies were 0.87 (95% CI: 0.81-0.92) and 0.97 (95% CI: 0.94-0.98), respectively. Meta-analysis of six topic studies revealed that LLM-assisted screening demonstrated a high discriminative ability, with SROC curve analysis yielding an area under the curve (AUC) of 0.97 (95% CI: 0.96-0.98) (Figure S7 in Appendix 1).

**S8.Post hoc meta-analysis of index results of LLM-assisted screening vs. conventional manual screening.**

To assess the impact of false-negative literature on the final conclusions, a separate post-hoc meta-analysis was conducted. This analysis compared the pooled effect sizes derived from LLM-assisted screening (including only true positives) with those from conventional screening (including both true positives and false negatives) for topic study 1, 2, 3, 5, and 6, respectively. Study 4 was not compared because the screening results from the two methods were consistent (Table S3 in Appendix 1). The results indicated comparable outcomes between the two methods in most topic studies (Figures S9-S12, S13, S16, and S17 in Appendix 1). Furthermore, the exclusion of false-negative articles did not substantially alter the overall conclusions of the corresponding topic studies in the majority of instances (Table S5 in Appendix 1). Notably, however, for outcomes on lymph node dissection (Study 3; Figure S14 in Appendix 1) and overall survival (Study 5; Figure S15 in Appendix 1), the exclusion of false negatives shifted the results from statistically significant positive effects to non-significant negative effects. An examination of the studies included in the study 3 and 5, focusing on the outcomes of lymph node dissection and overall survival, revealed significant heterogeneity and publication bias in the reported findings. This suggests that in meta-analyses demonstrating low heterogeneity and an absence of publication bias, the inclusion or exclusion of potential false-negative studies may not substantially impact the overall conclusions.

**References**

1. Oami T, Okada Y, Sakuraya M, et al. Efficiency and Workload Reduction of Semi-automated Citation Screening Software for Creating Clinical Practice

2. Gates A, Johnson C, Hartling L. Technology-assisted title and abstract screening for systematic reviews: a retrospective evaluation of the Abstrackr machine learning tool. *Syst Rev* 2018; 7: 45.Guidelines: A Prospective Observational Study. *J Epidemiol* 2024; 34: 380–386.

3. Van de Schoot R, de Bruin J, Schram R, et al. An open source machine learning framework for efficient and transparent systematic reviews. *Nat Mach Intell* 2021; 3: 125–133.

4. Gates A, Guitard S, Pillay J, et al. Performance and usability of machine learning for screening in systematic reviews: a comparative evaluation of three tools. *Syst Rev* 2019; 8: 278.
